# Effects of SSRIs on the spatial transcriptome of dorsal raphe serotonin neurons

**DOI:** 10.1038/s41380-026-03644-x

**Published:** 2026-05-15

**Authors:** Charlotta Henningson, Jakub Mlost, Iskra Pollak Dorocic

**Affiliations:** Science for Life Laboratory, Department of Biochemistry and Biophysics, Stockholm University, Stockholm, Sweden

**Keywords:** Neuroscience, Depression, Molecular biology

## Abstract

The serotonin system is the main therapeutic target for selective serotonin reuptake inhibitors (SSRIs) in treating depression, yet the mechanism of action of SSRIs remains incompletely understood. To investigate the molecular and transcriptional effects of SSRI administration on serotonin neurons, we performed spatial transcriptomics, a spatially resolved RNA-sequencing method in intact brain tissue. Mouse brain sections containing the dorsal raphe nucleus and adjacent midbrain structures were analyzed, revealing six distinct serotonergic subpopulations with unique molecular signatures and spatial distributions. Both acute and chronic fluoxetine treatment induced a large number of changes in gene expression in the dorsal raphe nucleus. Notably, *Htr1a* expression increased following acute treatment but decreased after chronic administration, supporting previous findings on serotonin transporter blockade effects on 5-HT1A autoreceptors. Gene enrichment and network analysis identified key pathways modulated by SSRI administration, including Ras, MAPK and cAMP signaling pathways as well as pathways involved in axonal guidance. Additionally, we observed treatment-dependent opposing transcriptional changes in neuropeptides, particularly Thyrotropin-releasing hormone (*Trh*) and Prodynorphin (*Pdyn*), with distinct spatial localization within the dorsal raphe nucleus. Collectively, our transcriptomic and in situ hybridization analyses reveal spatial and cell-type-specific heterogeneity in SSRI action within the dorsal raphe nucleus, providing new insights into the molecular basis of SSRI treatment effects.

## Introduction

Depression is a leading contributor to the global disability burden, with high lifetime prevalence, elevated risk of suicide, and critically increasing incidence [1]. Selective Serotonin Reuptake Inhibitors (SSRIs), which directly target the brain serotonin system, are the most commonly prescribed treatment [2, 3]. Despite their widespread clinical use for depression and comorbid conditions, the molecular and transcriptional responses to SSRI administration within serotonergic neurons and surrounding neural populations remain incompletely characterized. Understanding how SSRIs modulate gene expression in serotonin neurons and their local circuit environment within their precise anatomical context is essential for elucidating the cellular mechanisms of action of these drugs.

The serotonin system exhibits remarkable molecular and anatomical heterogeneity. Serotonergic neurons, located exclusively within the brainstem raphe nuclei, project throughout the brain to modulate diverse neurophysiological functions ranging from homeostatic regulation to complex mood and motivational states [4]. The dorsal raphe nucleus (DR), containing the majority of forebrain-projecting serotonin neurons, is embedded within complex neural circuits receiving inputs from, and projecting to, a large number of brain regions [5–8]. Previous research has identified molecular and functional distinctions among serotonergic subpopulations based on electrophysiological properties, projection patterns, and developmental origins [9–12], as well as separate roles in behavior [13, 14]. However, the lack of specific molecular markers limited the ability to systematically characterize these differences and their pharmacological responses. Single-cell RNA sequencing studies have begun to address this gap, by revealing distinct molecular signatures that define serotonergic subtypes [15–17]. The cellular diversity in the DR is further enhanced by the presence of other neuronal types, including dopaminergic, glutamatergic, and GABAergic neurons, as well as various neuropeptide-expressing cells [18, 19]. Comprehensive, unbiased spatial mapping of gene expression is therefore important to understand how molecular heterogeneity is organized across DR anatomy and how spatially distinct neuronal populations respond differentially to pharmacological interventions.

SSRIs directly act on serotonin neurons by rapidly blocking the serotonin transporter (SERT), elevating extracellular serotonin concentration within hours [20], yet clinical efficacy requires weeks of chronic administration [21]. This temporal discrepancy is thought to involve 5-HT1A autoreceptor desensitization: initial SSRI-induced elevation of synaptic serotonin activates these autoreceptors, resulting in decreased serotonin neuron firing and release [22, 23]. Chronic SSRI treatment leads to 5-HT1A autoreceptor desensitization and subsequent loss of feedback inhibition, ultimately increasing serotonin neuron activity [24–26]. While this model has been influential in explaining the delayed therapeutic onset of SSRIs, alternative mechanisms have also been proposed [27]. Antidepressants induce widespread transcriptional changes across multiple brain regions, including the prefrontal cortex, hippocampus, and amygdala [28–30]. However, previous studies have primarily focused on limited candidate genes in specific forebrain regions. Recent unbiased transcriptomic profiling revealed that chronic fluoxetine produces region-specific effects throughout the brain, with the most pronounced changes occurring in the DR [31, 32]. However, the specific effects on serotonin neurons, their spatial subtypes, and other cell types in the DR are unknown.

Here, we employed spatial transcriptomics coupled with multiplexed fluorescent in situ hybridization to comprehensively map the molecular architecture of the mouse DR and characterize its response to acute and chronic fluoxetine administration. We first established a spatially-resolved transcriptional atlas of the DR, identifying molecular markers that define distinct serotonergic subpopulations and their anatomical distributions. We then revealed dynamic transcriptional responses to acute versus chronic SSRI treatment across these neural populations. Our findings provide insight into the spatial and molecular heterogeneity of SSRI action within the DR, with implications for understanding the complex mechanisms underlying antidepressant action. The data is accessible through an interactive app available at https://st-ssri.serve.scilifelab.se/app/st-ssri.

## Methods

### Animals

All procedures using animals were approved by the Stockholm Ethics committee (Dnr 5966-2021). Mice were housed in individually ventilated GM500 cages (Tecniplast) under constant humidity (50–60%) and temperature (21 ± 2 °C) and with a constant (year-around) 12-h light/dark cycle. All experiments were carried out in adult C57BL/6 J (Charles River) male and female mice ranging in age from 8–12 weeks. All mice were group housed with a maximum of 5 mice per cage. For spatial transcriptomics experiments, 9 mice (5 male, 4 female) were used, for in situ hybridization 17 mice (9 females, 8 males) were used.

### Fluoxetine administration

Fluoxetine Hydrochloride (Sigma-Aldrich, F132) was dissolved in saline and stored at −20 °C until use. For administration, fluoxetine hydrochloride was diluted to 2 mg/ml and administered orally at 10 mg/kg, using soft gavage feeding tubes. This dose was selected based on both allometric scaling and pharmacokinetic validation. Using interspecies dose conversion [32–34], the 10 mg/kg mouse dose converts to 0.81 mg/kg human equivalent dose, falling within the clinical range of 0.33–1.33 mg/kg for depression treatment [35]. Importantly, this dose has been validated to achieve therapeutically relevant drug exposure: chronic administration of 10 mg/kg fluoxetine in mice produces steady-state plasma concentrations of 200–300 ng/ml [36], comparable to therapeutic human plasma levels (150–500 ng/ml), and achieves ~80% serotonin transporter occupancy in brain tissue [37, 38]. These pharmacokinetic parameters correlate with antidepressant-like behavioral effects emerging after 3–4 weeks of treatment [36, 39], mirroring the delayed therapeutic onset observed clinically.

Mice were assigned to three treatment groups (*n* = 3/treatment for spatial transcriptomics, *n* = 6/treatment for in situ hybridization). The acute treatment group received a single 10 mg/kg dose of fluoxetine, with brain tissue harvested 1.5 h post-administration. This timepoint was selected based on pharmacokinetic studies showing peak brain concentrations occur 1–2 h after oral administration [23, 40], and behavioral effects are detectable within 30 min in C57BL/6 J mice [41, 42]. The chronic treatment group received 10 mg/kg fluoxetine daily for 22 consecutive days. On day 22, brain tissue was harvested 5 h after the final dose to minimize acute pharmacological effects. The control group received a single gavage of saline with brain harvest at 1.5 h to match the acute treatment timeline.

### Tissue collection

Brain tissue was flash-frozen on dry ice and stored at −80 °C. Cryosections were cut at 10μm (spatial transcriptomics) or 20μm (RNAscope) and mounted on Visium slides or SuperFrost+ slides, respectively.

### Fluorescent in situ hybridization

Multiplexed RNAscope HiPlex (Advanced Cell Diagnostics) was performed following the manufacturer’s protocol for fresh frozen tissue, using eight probes: Slc6a4, Htr1a, Vip, Pdyn, Trh, Bdnf, Slc17a8, Gad2. Two-round imaging was performed on a Leica DM6B microscope (40x oil immersion) capturing five channels per round (DAPI, 488, 550, 650, 750 nm).

### Image analysis

Images were processed in Fiji (ImageJ 2.14.0) and QuPath v0.5.0 [43, 44]. BigWarp was used to align imaging rounds based on DAPI staining [45]. Cell segmentation employed StarDist [46] with the dsb2018_heavy_augment model (5.0μm expansion, 0.5 threshold). Regions of interest were selected to define the DR based on Slc6a4+ expression. Cell classification utilized QuPath’s object classifier trained on marker expression patterns. RNA puncta were quantified using subcellular detection with thresholds set above autofluorescence and negative controls.

### Spatial transcriptomics

Visium spatial gene expression assay (10x Genomics) was performed following manufacturer’s protocols. Briefly, the coronal sections were fixed before performing H&E staining and imaging. The tissue was then permeabilized enzymatically and the RNA was captured on the barcoded probes attached to the glass slide by the poly-A tail. Libraries were generated for each capture area and sequenced. Manual alignment of the H&E stained histology images to the expression spot grid was performed using the 10x Genomics Loupe Browser software (v. 5.1.0). The raw sequencing data files (FASTQ files) were processed using the 10x Genomics Space Ranger software (v. 1.3.0) using the mouse genome reference transcriptome version mm10 (GRCm38) 2020-A (July 7, 2020) provided by 10x Genomics.

### Analysis of Visium sequencing data

Data processing employed Seurat v4.3.0 in R [47]. Quality filtering excluded spots with <1000 genes/features or >24% mitochondrial content. Hemoglobin, mitochondrial, ribosomal, and sex chromosome genes were removed. Normalization used SCTransform with glmGamPoi, regressing mitochondrial and ribosomal percentages. Integration across capture areas employed reciprocal PCA (top 3000 features, 30 PCs) with control samples as reference.

#### Spatial cluster identification and selection

Clustering of the complete midbrain dataset was performed using unsupervised clustering with the Louvain algorithm (resolution=0.9), with UMAP visualization (20 PCs, 50 neighbors). The DR cluster was identified based on its molecular signature and its spatial correspondence with the anatomical location of the DR. All spots assigned to this cluster by the unsupervised clustering algorithm were included in subsequent DR-focused analyses. To resolve spatial and molecular heterogeneity within the DR, we performed focused subclustering on the DR cluster spots using the same approach (resolution=0.8, 8 PCs), to resolve serotonergic heterogeneity.

#### Differential expression and network analysis

Differential gene expression between conditions employed Wilcoxon rank-sum test (log2FC > 0.5, expression in >20% spots, FDR < 0.05). Protein-protein interaction networks were constructed using STRING database and visualized in Cytoscape v3.10.0 [48], with hub genes defined by degree >6. Functional enrichment utilized DAVID (Database for Annotation, Visualization, and Integrated Discovery) for KEGG (Kyoto Encyclopedia of Genes and Genomes) pathways and Gene Ontology (GO) biological processes [49, 50]. Neuronal activity signatures were assessed using NEUROeSTIMator v0.1 [51]. Expression patterns of DR-derived DEGs, including subsets annotated to KEGG pathways and GO biological processes, were evaluated within Visium DR spatial subclusters and across serotonergic subtypes from a published scRNA-seq dataset [16].

#### Cell type deconvolution

CytoSPACE v1.0.5 and SpaTalk (v1.0) [52, 53] mapped published scRNA-seq data [16] onto spatial transcriptomics spots, estimating 9 cells/spot based on VistoSeg analysis [54]. All available cell types were mapped, but only serotonin subtypes were isolated for visualization and further quantification.

### Statistical analysis

For RNAscope quantification, one-way ANOVA compared treatment groups followed by Holm-corrected pairwise t-tests. Spatial transcriptomics employed non-parametric tests with FDR correction for multiple comparisons. Significance threshold was *p* < 0.05. Sample sizes (3 mice/group for spatial transcriptomics, 6 mice/group for ISH) were based on prior spatial transcriptomics studies, with power to detect 1.5-fold expression changes.

## Results

### Spatial transcriptomic profiling of midbrain and DR

**Fig. 1:**
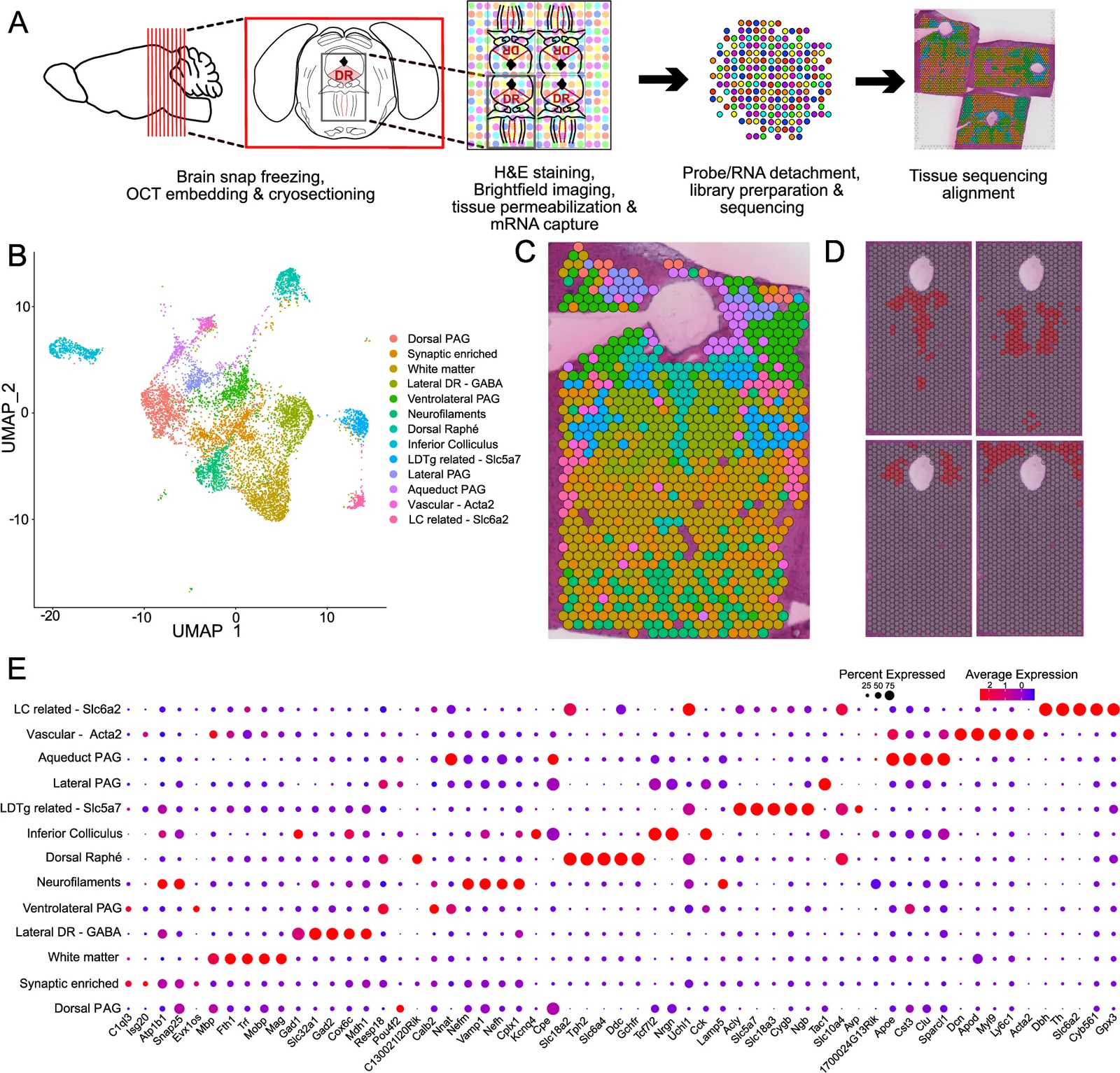
Spatial transcriptomic profiling reveals distinct molecular domains in the mouse midbrain and DR. **A** Experimental strategy: Coronal sections of mouse midbrain including the DR were cut and multiple sections were mounted on Visium slide capture areas, followed by RNA sequencing and tissue alignment. **B** UMAP visualisation of clusters based on the transcriptional signature of each spot, covering the whole midbrain section, revealed 13 molecular clusters. Acronyms in cluster names: PAG, periaqueductal gray; DR, dorsal raphe; LDTg, laterodorsal tegmental nucleus; LC, locus coeruleus. **C** Representative midbrain tissue section with overlaid sequencing spots, color represents molecular cluster in (B). **D** Visualization of four anatomically distinct spatio-molecular clusters, including DR (top left), lateral DR (top right), lateral PAG (LPAG, bottom left) and dorsal PAG (bottom right). **E** Dot plot for each molecular cluster showing expression level of individual genes. Diameter of the dot corresponds to the percentage of spots in the cluster expressing the gene, color indicates average expression of the gene.

Using unbiased and untargeted spatial transcriptomics, we profiled gene expression in coronal brain sections containing the DR and neighboring regions, in adult mice (Fig. 1A). We detected, on average, 3892 genes and 10,239 reads per spot (3892 ± 1133 unique genes, 10,239 ± 4978 reads). The complete dataset after quality control contained information on the expression of 30,413 unique genes across 8369 spots. Shared Nearest Neighbor (SNN) clustering of gene expression across the whole brain section revealed 13 distinct clusters (Fig. 1B). We did not observe batch nor sex mediated effects on clustering (Fig. S1). Aligning the molecular clusters with spatial location in brain tissue largely recapitulated anatomical brain regions, but also provided a higher-resolution delineation within the same brain region (Fig. 1C). For example, the periaqueductal gray (PAG) can be subdivided into the dorsal, lateral, ventrolateral and aqueductal PAG subregions based on distinct molecular gene expression profiles. Spatial sequencing also clearly reveals the DR anatomical cluster, as well as a cluster flanking the DR bilaterally with distinct molecular signatures (Fig. 1D).

Unsupervised clustering defined the DR cluster which expresses canonical serotonin markers, relating to serotonin synthesis, reuptake, release and regulation. These include: *Tph2* (Tryptophan hydroxylase 2), *Slc6a4* (Serotonin transporter, SERT), *Slc18a2* (Vesicular monoamine transporter 2, VMAT2), *Ddc* (Dopa decarboxylase), and *Gchfr* (GTP Cyclohydrolase I feedback regulatory protein) that is involved in post-translational regulation of the serotonin synthesis pathway [55] (Fig. 1E). Another molecular cluster (lateral DR-GABA), flanks the DR bilaterally and is marked by *Gad2* expression. Overall, the spatial sequencing approach revealed molecular markers for midbrain anatomical structures, and reliably identified the DR based on serotonin-associated gene expression. The subsequent analysis focuses on the DR cluster, in order to define spatial subpopulations of serotonin neurons, and determine the effects of acute and chronic fluoxetine treatment.

### Spatial subpopulations within DR serotonin system

The DR exhibits complex molecular heterogeneity across diverse cell populations, with single-cell RNA sequencing (scRNA-seq) studies revealing distinct serotonergic subpopulations with unique transcriptomic signatures [15–17]. However, scRNA-seq inherently lacks spatial context, precluding direct assessment of how molecular diversity relates to anatomical organization within the DR. While previous efforts have bridged this gap by mapping molecularly-defined subtypes to anatomical locations through in situ hybridization of select candidate genes, these approaches remain fundamentally limited by their reliance on predetermined gene sets. No fully unbiased analysis directly linking complete transcriptomic profiles to anatomical location in tissue has been performed in the DR.

**Fig. 2:**
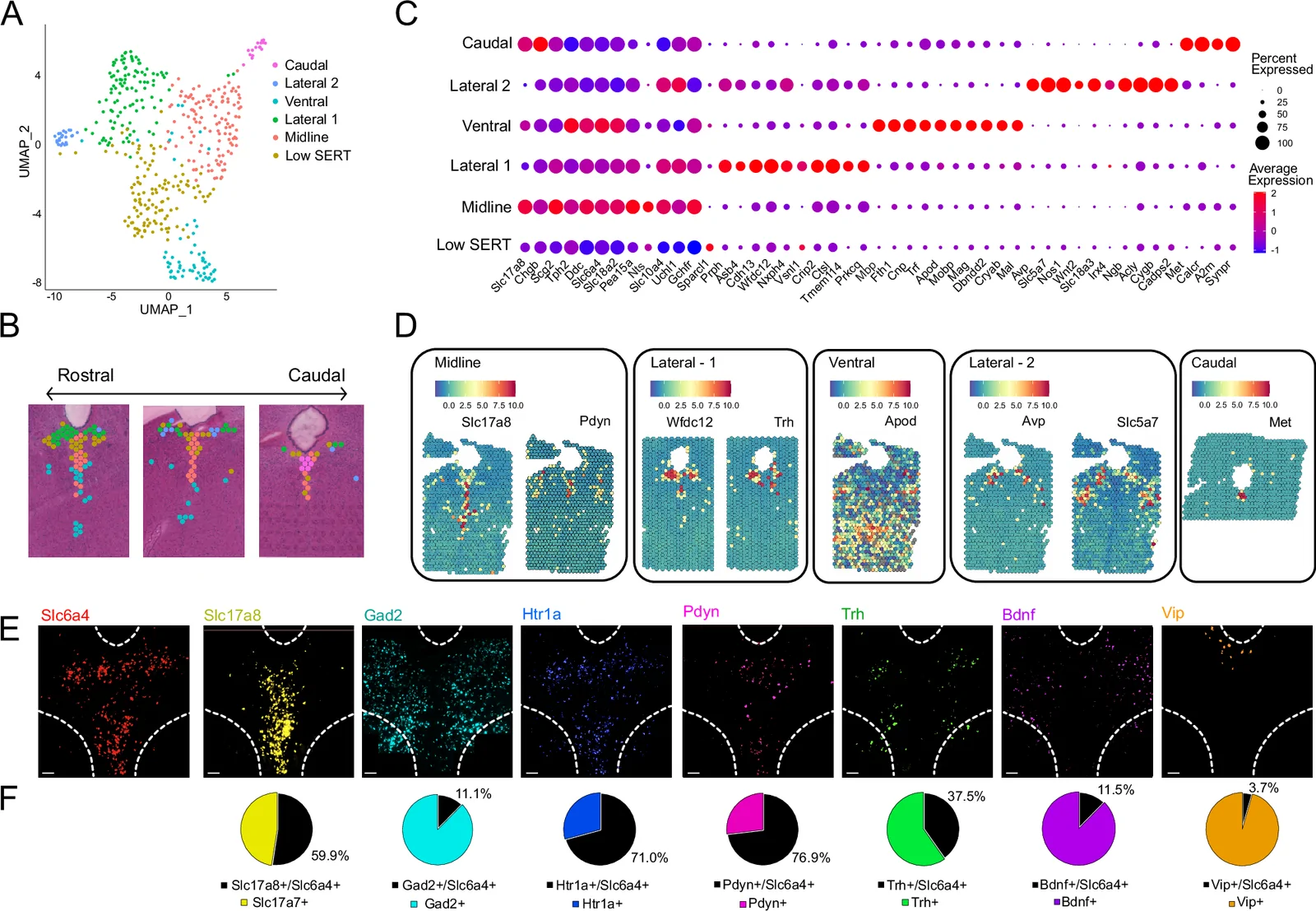
Spatio-molecular mapping of DR reveals specific spatial markers. **A** UMAP visualisation based on the transcriptional signature of each DR cluster spot, cluster names refer to the spatial location or molecular identity. **B** Molecular clusters from A overlaid with tissue section, showing spatial localization of each cluster in DR and rosto-caudal expression patterns. **C** Dot plot showing expression of 10 cluster marker genes per cluster. Diameter of the dot corresponds to the percentage of spots in the cluster expressing the gene, color indicates average expression of the gene. **D** Spatial heatmap of gene expression in DR for selected marker genes. Example of DR-specific markers (e.g. midline-*Slc17a8*, *Pdyn*) and non-DR-specific marker genes (ventral-*Apod*). **E** In situ hybridization in DR (area within broken white border) with 8 probes: *Slc6a4*, *Gad2*, *Slc17a8*, *Htr1a*, *Pdyn*, *Trh*, *Bdnf*, *Vip*, each displaying a distinct single-cell distribution pattern within DR. Scale bar = 100μm. **F** Quantification of the co-expression of each marker gene from (E) with serotonin marker *Slc6a4* (SERT).

Spatial transcriptomic analysis revealed 6 distinct molecular subclusters within the DR, each showing specific anatomical distributions along the rostro-caudal axis (Fig. 2A, B). These clusters demonstrated unique spatial organization: one midline, one ventral, one caudal, two lateral clusters, and one cluster with reduced serotonergic marker expression (Fig. 2C). The anatomical serotonergic clusters exhibit strong molecular identities characterized by distinct marker gene expression, while the low serotonergic cluster is defined by weak expression of canonical serotonergic markers, suggesting it may contain fewer serotonin-producing neurons relative to other DR subregions, or alternatively, neurons with low expression of serotonin markers. We identified cluster-specific marker genes, with several showing DR-restricted expression patterns: midline (*Slc17a8, Pdyn*), caudal (*Met*), and lateral cluster 1 (*Wfdc12, Trh*) (Fig. 2D). Other markers, including ventral cluster-associated *Apod* and lateral cluster 2-associated *Avp* and *Slc5a7*, showed broader expression patterns extending beyond the DR. Notably, lateral cluster 2 exhibited a distinct molecular profile, likely influenced by its proximity to cholinergic neurons of the rostral lateral dorsal tegmental nucleus, as supported by *Slc5a7* expression. The ventral cluster corresponds anatomically to the location of the median raphe nucleus (MR), however, the predominant marker genes are associated with oligodendrocytes, as this cluster contains a higher proportion of glial cells compared to other subclusters (Fig. 2C, Fig. S3F).

To validate our spatial transcriptomics findings at single-cell resolution, we performed multiplexed in situ hybridization (ISH) for key marker genes in the DR (Fig. 2E, F). The midline cluster is defined by the expression of *Slc17a8*, the gene coding for Vesicular Glutamate Transporter 3 (VGLUT3). This neural subpopulation has been extensively characterized, and has been shown to contain serotonin neurons that co-express VGLUT3 and are able to co-release glutamate in several forebrain regions [56–58]. We found that approximately half of *Slc17a8* neurons in the DR express *Slc6a4*, the gene coding for SERT (59.9% +/- 8.3% of *Slc17a8* cells express *Slc6a4*, mean ± SD, *n* = 1979 *Slc17a8* neurons, *n* = 6 mice) consistent with previous characterizations of this co-releasing population [18]. The GABAergic marker gene *Gad2*, which codes for Glutamate Decarboxylase 2, showed widespread expression throughout the DR and adjacent regions. A minority of DR *Gad2* cells co-expressed *Slc6a4* (11.1% +/- 5.1% of *Gad2* cells express *Slc6a4*, *n* = 3718 *Gad2* neurons, *n* = 5 mice). Analysis of the serotonin autoreceptor *Htr1a* revealed extensive co-expression with *Slc6a4* (71.0% ± 10.2% of *Htr1a* cells express *Slc6a4*, *n* = 3114 *Htr1a* neurons from 6 mice), confirming its predominant but not exclusive association with serotonergic neurons.

Our spatial transcriptomics analysis identified additional molecular markers with distinct spatial distributions in the DR. *Pdyn*, encoding the neuropeptide prodynorphin, showed strong midline expression and substantial co-localization with serotonergic neurons (76.9% +/- 6.5% of *Pdyn* cells express *Slc6a4*, *n* = 1235 *Pdyn* neurons, *n* = 6 mice). *Trh* (thyrotropin-releasing hormone) exhibited a specific expression pattern in the caudal and ventral lateral wings of the DR, with moderate serotonergic overlap (37.5% +/- 16.8% of *Trh* cells express *Slc6a4*, *n* = 483 *Trh* neurons, *n* = 5 mice). *Bdnf* (brain derived neurotrophic factor), linked to the regulation of stress and development of mood disorders [59], showed predominantly lateral expression with limited serotonergic co-expression (11.5% +/- 6.9% of *Bdnf* cells express *Slc6a4*, *n* = 2709 *Bdnf* neurons, *n* = 5 mice). *Vip* (vasoactive intestinal peptide) was concentrated in the dorsal DR along the aqueduct, with minimal serotonergic overlap (3.7% +/- 3.0% of *Vip* cells express *Slc6a4*, *n* = 356 *Vip* neurons, *n* = 6 mice). A portion of these DR Vip-expressing neurons have been previously characterized as co-expressing the dopamine transporter DAT [60]. We integrated our findings into a comprehensive molecular map showing the spatial distribution and serotonergic co-expression patterns of each marker along the rostro-caudal axis (Fig. S2). These results reveal distinct molecular signatures that define spatially segregated neuronal populations within the DR.

Next, we integrated our spatial dataset with a previously published scRNA-seq DR dataset [16], to map the five identified molecular serotonergic subtypes (5-HT-I through V) onto our spatial transcriptomics data using CytoSPACE (Fig. S3A). CytoSPACE is a computational approach to integrate scRNA-seq and spatial transcriptomics data by assigning single cells to spatial locations based on gene expression similarity to determine cell type composition of each spot [52]. This scRNA-seq dataset was selected because it contains multiple different cell types present in the DR, including non-neuronal types. Comprehensive cell type representation is essential for accurate integration and spatial mapping onto spatial transcriptomics data. Analysis revealed substantial spatial overlap between certain subclusters and subpopulations, particularly evident between 5-HT-V and the Caudal subcluster (Fig. S3A, B) Analysis of top spatial cluster gene expression patterns demonstrated distinct molecular signatures across serotonergic subpopulations (Fig. S3C, D). The 5-HT-V subtype and Caudal spatial cluster exhibited concordant expression profiles, characterized by expression of *Met*, *Calcr*, and several other genes. The 5-HT-IV subtype aligned with the Midline spatial cluster, sharing *Slc17a8* expression while showing minimal expression of Lateral 1 marker genes. Similarly, 5-HT-I subtype and the Lateral 1 spatial cluster demonstrated shared expression of multiple markers, including *Prph*, *Cdh13*, and *Ctsl*. In contrast, subpopulations 5-HT-II and III displayed mixed expression patterns, incorporating markers characteristic of both midline and lateral populations. Quantitative spatial distribution analysis revealed preferential localization patterns, with 5-HT-II predominantly present in Lateral 1 (56% ± 13.3% of mapped 5-HT-II cells, mean ± SD, *n* = 37 of 66 neurons) and 5-HT-III showing enrichment in Midline regions (71% ± 4.5% of mapped 5-HT-III cells, mean ± SD, *n* = 17 of 24 neurons) (Fig. S3E). Although neurons represent the predominant cell type within each of the DR subclusters, other cell types, including astrocytes and oligodendrocytes, are also present to varying extents across the clusters (Fig. S3F). These findings demonstrate that while serotonergic subtypes exhibit clear spatial preferences, each DR spatial cluster represents a distinct microenvironment characterized by specific combinations of 5-HT subtypes.

### Effects of acute and chronic SSRI treatment on DR serotonin system

SSRIs directly target serotonergic neurons by blocking SERT, yet their effects on gene expression within these neurons are not known. Previous transcriptional studies have focused on downstream brain regions innervated by serotonergic projections, leaving a critical gap in our understanding of SSRI action at its primary site of effect. To address this, we employed spatial transcriptomics to analyze gene expression changes in the DR following fluoxetine administration. Given the well-documented temporal disparity between SSRI administration and therapeutic efficacy, we examined both acute and chronic treatment conditions to capture the dynamic transcriptional landscape underlying this therapeutic delay.

**Fig. 3:**
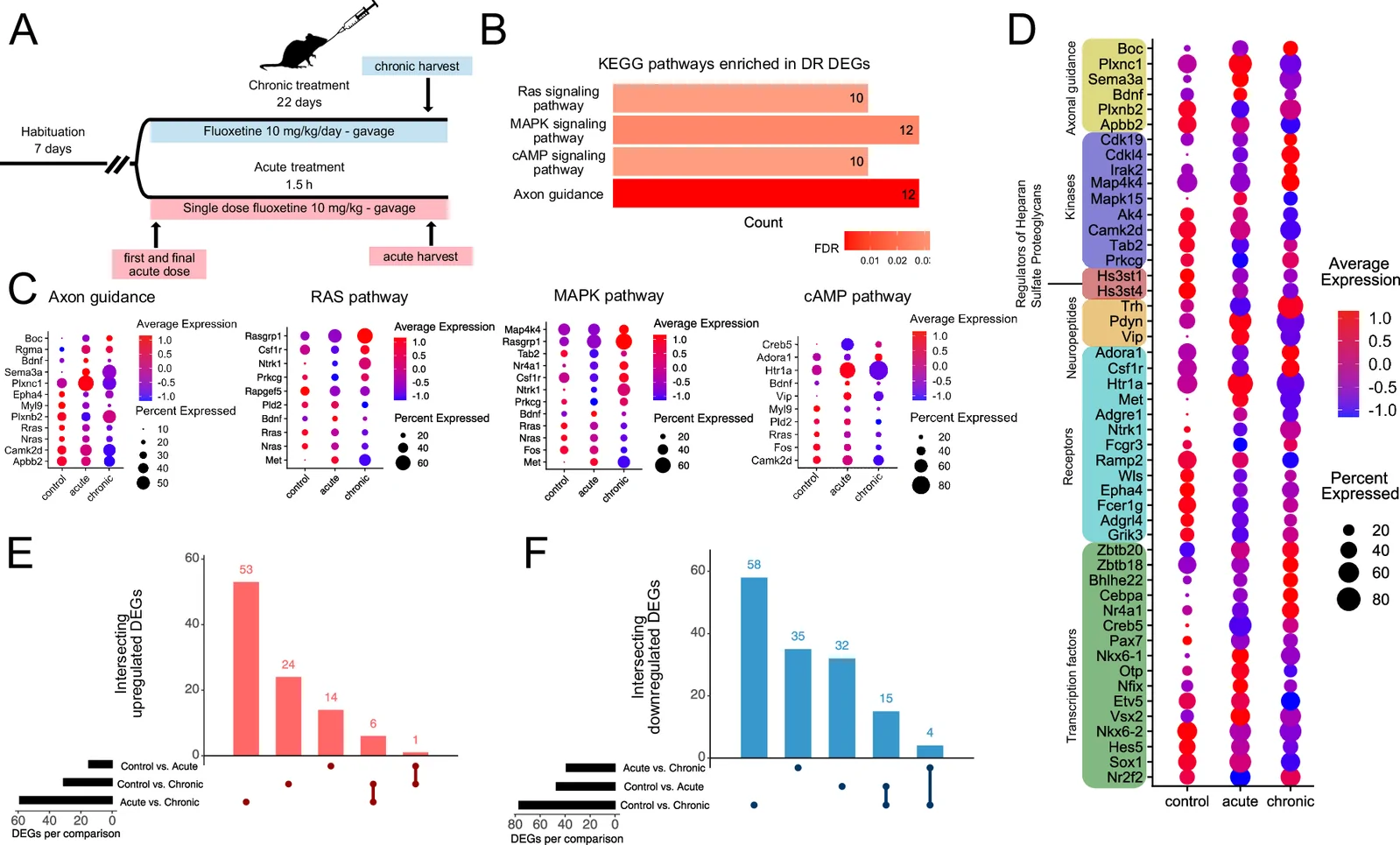
Acute and chronic SSRI treatment elicits distinct gene expression changes in the DR. **A** Schematic representation of the pharmacological experiment. **B** Gene enrichment analysis in the KEGG database of DEGs in the DR. Number in the bar represents the number DEGs expressed in each pathway, observed across all treatment conditions. **C** Dotplot of expression profiles of single DEGs in the DR, categorized by the involvement in specific KEGG pathways. **D** List of DEGs in the DR categorized by function from the Mouse Genome Informatics database and their expression levels across control, acute and chronic treatment conditions. **E** Upset plot of upregulated genes in the DR, between treatment conditions. **F** Upset plot of downregulated genes in the DR, between treatment conditions.

Three treatment protocols using gavage administration were administered to healthy, naïve mice: acute (single dose fluoxetine, 10 mg/kg), chronic (22 days daily administration, 10 mg/kg), and control (single dose of saline) (Fig. 3A). Our analysis identified a range of differentially expressed genes (DEGs) between the control, acute and chronic treatment protocols in both the whole midbrain tissue clusters (Fig. S4A) and the DR clusters (Fig. 3B–D, Fig. S4B, C, Supplemental Table 1). Focusing specifically within the DR, acute and chronic SSRI administration resulted in DEGs enriched in several important signaling pathways and biological processes, including: Ras, MAPK, cAMP and axon guidance pathways (Fig. 3B). Chronic treatment led to widespread downregulation of axon guidance genes, most notably *Plxnc1*, which encodes a member of the plexin family and regulates axon guidance, cell motility and migration [61] (Fig. 3C). Conversely, chronic treatment upregulated *Rasgrp1*, an activator of the Erk/MAP kinase cascade. The serotonin autoreceptor gene *Htr1a* showed biphasic regulation, with upregulation following acute treatment and subsequent downregulation after chronic administration, consistent with previously described SSRI-induced autoreceptor adaptation [24–26]. Similarly, the immediate early transcription factor *Fos* used as a proxy for neural activity, is downregulated over chronic treatment (Fig. 3C). These findings reveal broad temporal dynamics in gene expression across multiple functional pathways during SSRI treatment (Fig. 3D, Fig. S4C).

We investigated how many of the DEGs are uniquely modulated in each treatment condition by analyzing the degree of overlap between the DEGs in control, acute and chronic fluoxetine administration condition (Fig. 3E, F). From a total of 232 DEGs identified, 144 showed downregulation and 98 showed upregulation across all different treatment conditions. Comparing the treatments, the number of downregulated DEGs in the control vs chronic condition is highest, while the number of upregulated DEGs is highest in the acute vs chronic condition. Each treatment comparison shows a large number of unique upregulated and downregulated DEGs. Interestingly, very few up- and down-regulated DEGs are shared between all three treatment conditions. The three genes showing differential expression in all three conditions are *Pdyn*, *Esam*, and *Nptx2* (Supplemental Table 1).

**Fig. 4:**
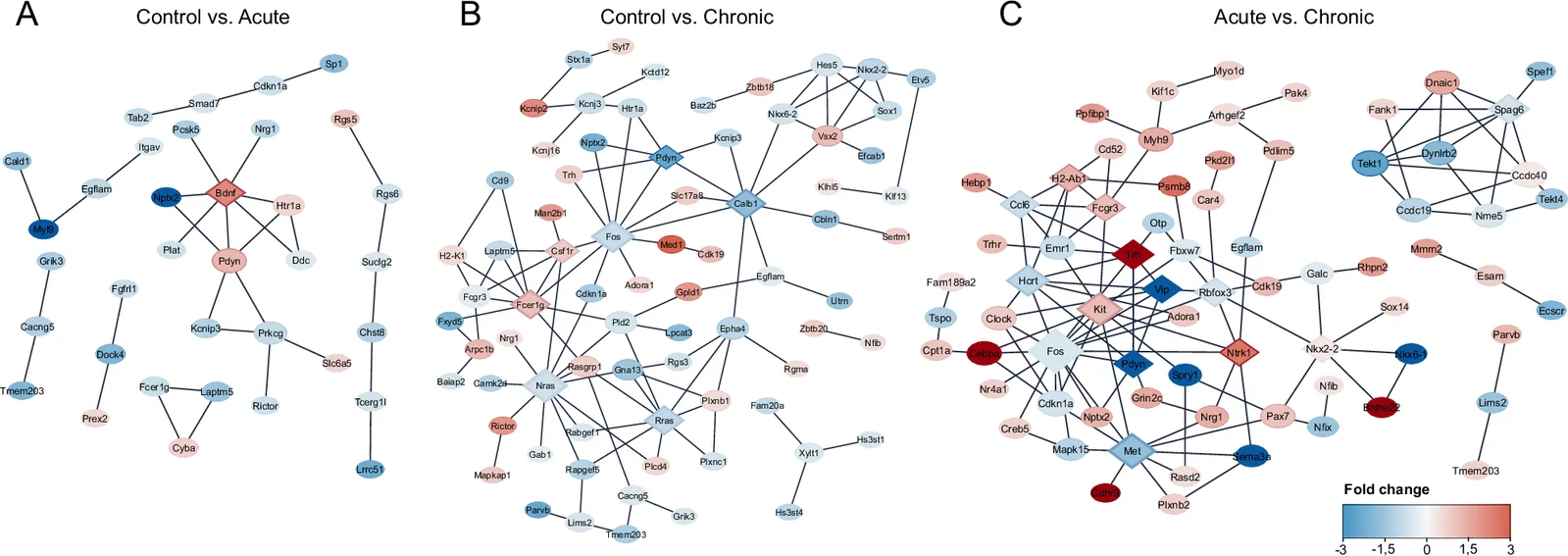
Network analysis of DR DEGs identifies key regulatory hubs across SSRI treatment. Protein-protein interaction network based on DR DEGs in STRING database for visualisation of changes in each comparison condition: **A** control vs. acute, **B** control vs. chronic and **C** acute vs. chronic. Hub genes are marked in a diamond node (♦). Red color denotes upregulation, blue denotes downregulation in comparison to control (A-B) or acute (C) group.

In order to find key factors among DEGs, we performed network analysis of protein-protein interactions (Fig. 4). Following acute SSRI treatment, *Bdnf* was the only hub gene detected, and was among the top three upregulated genes along with *Pdyn* and *Rgs5*, whereas the top three downregulated genes were *Myl9, Nptx2* and *Adgrl4* (Fig. 4A). Following chronic SSRI treatment, we found 7 hub genes: *Nras, Fos, Calb1, Rras, Fcer1g, Pdyn* and *Csf1r* (Fig. 4B). Among the top three upregulated genes were *Med1, Fcer1g, Csf1r*, whereas top three downregulated genes were *Pdyn, Nptx2* and *Fxyd5* (Fig. 4B). When comparing acute vs chronic SSRI treatment, we found 10 hub genes: *Fos, Met, Kit, Hcrt, Rbfox3, Trh, Ntrk1, Vip, Pdyn, Nkx2-2* (Fig. 4C). Among the top three upregulated genes were *Socs4, Bmp1* and *Bhlhe22*, whereas top three downregulated genes were *Vip, Tnnt1* and *Nkx6-1* (Fig. 4C). Overall, more upregulation was seen in acute vs chronic treatment compared to the other treatment comparisons. Interestingly, the two neuropeptide genes, *Pdyn* and *Trh*, showed opposite dynamic regulation over the time course of SSRI treatment. *Pdyn* was upregulated after acute and downregulated after chronic treatment, compared to the baseline. By contrast, *Trh* initially trended down, and was upregulated after the course of treatment (Fig. 3D).

Analysis of neuronal activity patterns revealed distinct temporal responses to SSRI treatment across DR subregions (Fig. S4D). Using NEUROeSTIMator [51], a bioinformatic approach that generates normalized activity scores based on the expression of 22 immediate early genes, we observed region-specific alterations in neuronal activity. Chronic SSRI administration resulted in significantly reduced overall activity in the DR compared to control conditions. This reduction in activity was specifically localized to the midline subcluster, while the lateral subcluster maintained baseline activity levels. Notably, this activity difference was only observed when comparing acute versus chronic treatment conditions. Analysis of the aggregate dataset showed no significant treatment effect on overall neuronal activity.

To determine whether SSRI-induced transcriptional changes occur in specific spatial or molecular clusters, we mapped the expression of the 232 DR-identified DEGs to the DR spatial clusters as well as the scRNA-seq derived serotonergic subtypes 5-HT-I through 5-HT-V [16] (Fig. S5, Supplemental Table 2). This analysis revealed remarkably broad expression of DEGs across different spatial clusters, with 184 DEGs expressed in all six spatial clusters. A further 17 DEGs were expressed in five of the spatial clusters, and there is minimal cluster-restricted expression (Fig. S5A). This indicates that SSRI-induced transcriptional changes occur broadly throughout the anatomical extent of the DR. Focusing on molecular 5-HT-I through -V subtypes, a total of 211 of the 232 DEGs were expressed, with 94 DEGs being shared across all five subtypes (Fig. S5B). This suggests that many core SSRI-responsive transcriptional programs are shared among serotonergic subtypes. However, there is heterogeneity in levels of expression of the DEGs and cellular prevalence among the serotonergic subtypes, at the single gene level. For example, even though they are detected in multiple subtypes, *Pdyn* is more robustly expressed in the 5-HT-III subtype, while *Trh* is mostly restricted to 5-HT-I subtype (Fig. S5C, D). Subtype 5-HT-I uniquely expresses two DEGs: *Tekt4* and *Cebpa*, while subtype 5-HT-II uniquely expresses *Ucma* and *Cd5*.

We further analyzed the distribution and directionality of DEGs within KEGG pathways and GO Biological Processes across our six spatial clusters and the previously described five serotonergic subtypes (Fig. S6, Supplemental Table 2, 3). Analysis of four major signaling pathways (Axon guidance, cAMP signaling, MAPK signaling and Ras signaling) revealed relatively uniform pathway representation across spatial clusters, with downregulated DEGs being more prevalent than upregulated DEGs (Fig. S6A, B). Examination of serotonergic subtypes revealed that the 5-HT-V subpopulation consistently showed the most restricted pathway engagement, with predominantly downregulated genes in cAMP and Ras signaling pathways (Fig. S6C, D). In contrast, subtypes 5-HT-I through -IV showed mixed up- and downregulation. Notably, *Htr1a* and *Slc6a5*, which codes for glycine transporter 2, are unique in showing bidirectional regulation (acute upregulation, chronic downregulation) across all five subtypes (Supplemental Table 1 and 3). The low number of expressed DEGs associated with 5-HT-V is likely influenced by the limited number of cells profiled in this molecular subtype [16].

Examining individual DEGs revealed important examples of non-serotonergic regulation. For instance, *Bdnf* was identified as significantly upregulated following acute SSRI treatment in spatial transcriptomics analysis and served as a hub gene in network analysis (Fig. 4A). However, analysis of the scRNA-seq dataset [16] showed that *Bdnf* falls below the 10% expression threshold in all profiled subtypes. This was confirmed by ISH, which demonstrated that only 11.5% ± 6.9% of *Bdnf*-expressing cells co-express the serotonergic marker *Slc6a4* (Fig. 2). Together, these findings indicate that SSRI-induced *Bdnf* upregulation occurs predominantly in non-serotonergic cells within the DR, demonstrating that transcriptional responses to serotonin transporter blockade extend beyond the directly targeted cell population to engage neighboring neurons and cell types.

In summary, temporal analysis of SSRI administration revealed extensive transcriptional remodeling within the DR, with the most pronounced differences emerging between acute and chronic treatment conditions. These changes encompassed individual genes, molecular pathways, and region-specific activity patterns across DR subdomains. The transition from acute to chronic fluoxetine treatment was characterized by the largest number of differentially expressed genes, affecting both up- and down-regulated transcriptional programs and associated signaling networks.

### Dynamic gene expression changes of DR neuropeptides in acute and chronic SSRI treatment

**Fig. 5:**
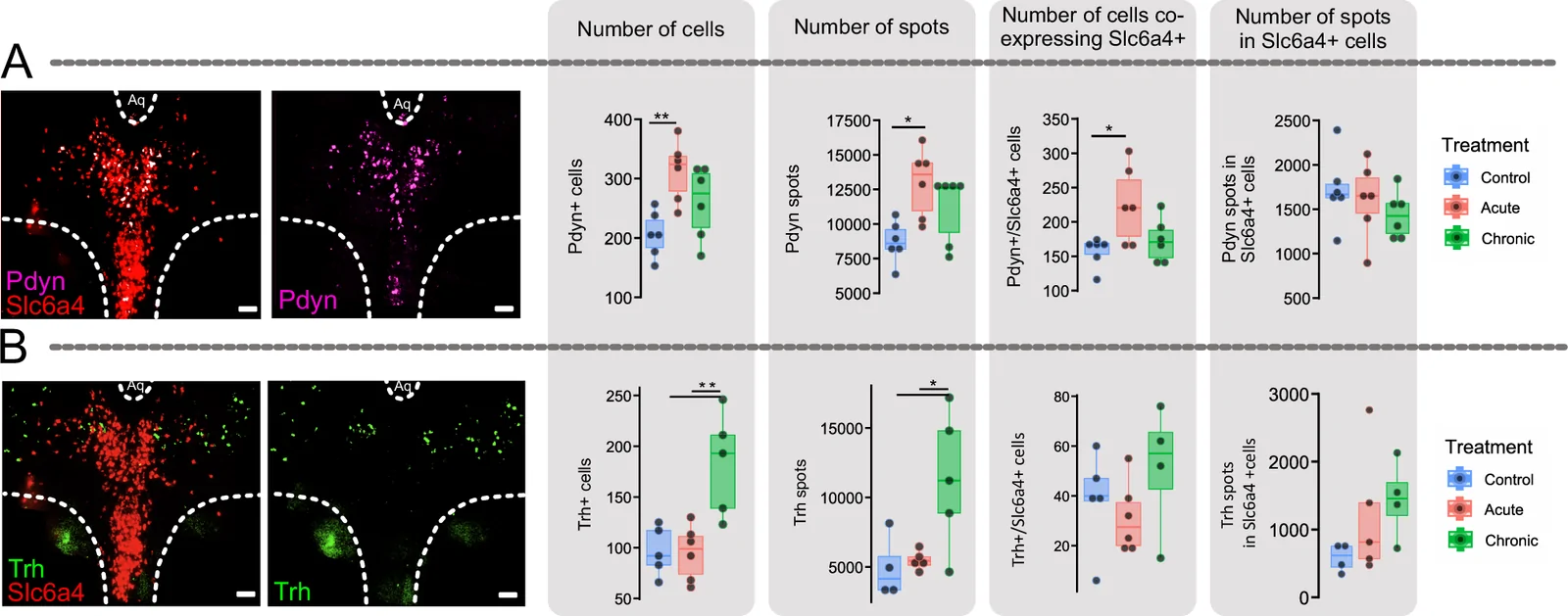
Dynamic gene expression changes of Pdyn and Trh in DR following acute and chronic SSRI treatment. **A** Representative images of in situ hybridization of *Pdyn* and *Slc6a4* co-expression and *Pdyn* in the DR. Box plots comparing treatment groups: sum of *Pdyn*+ cells per section (one-way ANOVA, F(2,15) = 6.64, p = 0.009**. Pairwise t-test with Holms correction; Acute vs Control, p = 0.0072**), sum of *Pdyn* spots (transcripts) per section (one-way ANOVA, F(2,15) = 5.735, p = 0.014*. Pairwise t-test with Holms correction; Acute vs Control, p = 0.0125*), sum of cells co-expressing *Pdyn* and *Slc6a4* per section (one-way ANOVA, F(2,15) = 5.004, p = 0.022*. Pairwise t-test with Holms correction; Acute vs Control, p = 0.0255*), and sum of *Pdyn* spots in *Slc6a4* cells. Control n = 6, acute n = 6, chronic n = 6 mice per group. **B** Representative images of in situ hybridization of *Trh* and *Slc6a4* co-expression and *Trh* in the DR. Box plots comparing treatment groups of sum of *Trh* cells per section (one-way ANOVA, F(2,13) = 10.29, p = 0.002**. Pairwise t-test with Holms correction; Acute vs Chronic, p = 0.00132**; Chronic vs Control, p = 0.00418**, control n = 5, acute n = 6, chronic n = 5 mice per group), sum of *Trh* spots (transcripts) per slice (one-way ANOVA, F(2,11) = 5.79, p = 0.019*. Pairwise t-test with Holms correction; Acute vs Chronic, p = 0.039*; Chronic vs Control, p = 0.039*, control n = 4, acute n = 5, chronic n = 5 mice per group), sum of cells co-expressing *Trh* and *Slc6a4* per section (control n = 5, acute n = 6, chronic n = 4 mice per group) and sum of *Trh* spots in *Slc6a4* cells (control n = 4, acute n = 5, chronic n = 4 mice per group). Dots represent individual animals, p-values shown follow p < 0.05 = *, p < 0.01 = **, p < 0.001 = ***. Scale bar = 100μm. Boxplots show the data distribution in each group, horizontal line denotes median.

Transcriptomic analysis revealed opposing regulation of *Pdyn* and *Trh* during SSRI treatment. To confirm these findings, we performed ISH on DR tissue after either acute or chronic fluoxetine treatment as well as with vehicle control. We found that acute administration increased both the number of *Pdyn*-expressing cells and total *Pdyn* mRNA spots in the midline DR (Fig. 5A). This acute upregulation was partially reversed following chronic treatment. Acute treatment also increased the number of neurons co-expressing *Pdyn* and *Slc6a4*. ISH analysis of *Trh* expression also corroborated our transcriptomic findings: chronic treatment increased both *Trh*-expressing cells and total *Trh* mRNA spots in the lateral DR (Fig. 5B). We observed no changes in *Trh* expression following acute treatment, nor in the number of neurons co-expressing *Trh* and *Slc6a4*. These findings demonstrate distinct regulatory mechanisms for *Pdyn* and *Trh*: SSRI-induced changes in *Pdyn* expression occur in serotonergic neurons, while *Trh* regulation appears independent of its co-expression with serotonin. Furthermore, ISH corroborated the spatial segregation of *Pdyn* and *Trh* within the DR, with *Pdyn* expression restricted to the midline and *Trh* to the lateral regions of DR.

The gene *Slc17a8* (VGLUT3) has been speculated to play a role in the treatment effect of SSRIs [62]. We sought to clarify whether the expression levels of *Slc17a8* alone, or in cells co-expressing *Slc6a4*, are affected by SSRI treatment and found no evidence of either (Fig. S7D). Though identified as DEGs in the transcriptomic analysis, ISH revealed no significant treatment effects upon *Htr1a*, *Bdnf*, or *Vip* expression in the DR, although *Bdnf* shows a trend following the transcriptomics findings (Fig. S7). Additionally, we investigated potential sex-specific effects of acute and chronic SSRI administration. The treatment-dependent regulation of *Pdyn* and *Trh* expression was consistent across male and female brains, while other markers showed no treatment effects in either sex. Overall, ISH analysis revealed no statistically significant sex-specific differences in the expression of *Pdyn*, *Trh*, *Slc17a8*, *Gad2*, *Vip*, *Bdnf*, and *Htr1a* across control, acute, or chronic treatment conditions (Fig. S8).

## Discussion

Through unbiased spatial transcriptomics, we identified and mapped region-specific markers across the DR. Consistent with previous immunohistochemical and transcriptomic studies, we detected *Slc17a8* enrichment in midline serotonergic neurons, *Pdyn* in midline domains, *Met* in caudal regions, and *Trh* in lateral DR [15–17, 19, 63, 64]. Our spatial transcriptomic approach extends these findings by simultaneously profiling the entire transcriptome, enabling identification of additional markers and assessment of how these spatially segregated populations respond differentially to SSRI treatment.

Comparison of acute and chronic treatment conditions revealed extensive transcriptional changes. Gene enrichment and network analysis identified key modulated pathways among the 232 differentially expressed genes in the DR, with 10–12 genes annotated to each of the Ras, MAPK, and cAMP signaling cascades, as well as axonal guidance pathways. SSRI-associated deficits in axonal guidance have been documented, particularly during development [65], and chronic fluoxetine treatment reduces both the density and morphology of serotonergic projections to the PFC and hippocampus [66, 67]. Our findings show that chronic treatment decreased expression of several axonal guidance genes, including *Plxnc1*. Pathway-level analysis revealed that SSRI-induced transcriptional changes engage major signaling cascades (Ras, MAPK, cAMP) and developmental processes broadly across DR spatial clusters and serotonergic subtypes, with minor spatial and molecular heterogeneity in the magnitude and directionality of responses. The predominance of downregulated over upregulated pathway-related DEGs suggests that chronic SSRI treatment may constrain or stabilize certain neuroplastic processes within the DR. Network analysis identified multiple Ras pathway regulators as hub genes. Changes in these hub genes and other highly regulated genes including *Bdnf*, *Csf1r*, *Nras*, *Rras*, *Fos*, *Met*, *Kit*, *Myl9*, and *Ntrk1* align with the neurotrophic hypothesis of antidepressant action [68].

Our integrative analysis revealed specific spatial and molecular heterogeneity in SSRI-induced transcriptional changes across the DR. While the majority of DR-derived DEGs are shared across all molecular serotonergic subtypes, we also identified subtype-specific regulation, including *Pdyn* in 5-HT-III (corresponding to the Midline spatial cluster) and *Trh* in 5-HT-I (corresponding to the Lateral-1 spatial cluster). This heterogeneity in DEG expression patterns across serotonergic subtypes and spatial domains indicates that SSRI action can differentially engage distinct serotonergic populations within the DR microenvironment. However, future studies using single-cell or single-nucleus RNA-seq following SSRI treatment will be necessary to fully resolve cell-type-specific transcriptional programs and their contributions to antidepressant action. Furthermore, our integration with the Huang et al. [16] dataset, selected for its multi-cell-type composition, may underestimate subtype heterogeneity compared to more granular scRNA-seq serotonergic classifications [15, 17].

Our analysis revealed significant changes in neuropeptide expression, potentially underlying direct behavioral effects of SSRIs. Of particular interest was the regulation of *Pdyn*, which encodes prodynorphin, an opioid peptide that activates kappa opioid receptors (KOR). While KOR activation produces analgesia, it also induces dysphoria and depressive-like behaviors, distinct from other opioid receptor subtypes [69]. *Pdyn* expression occurs in multiple limbic regions, including the amygdala, hippocampus, nucleus accumbens, and hypothalamus, and has recently been identified in a subpopulation of DR serotonin neurons [15–17]. Studies using the forced swim test have shown that KOR disruption reduces depression-like behavior [70], supporting KOR signaling’s pro-depressive role. Although KOR signaling has been implicated in stress-induced depressive symptoms through its interaction with the dopaminergic system, its relationship with serotonergic function in mood regulation remains less understood. DR KOR signaling is dysregulated by stress [71], and two distinct DR-*Pdyn* projections have been linked to addiction: DR *Pdyn*-expressing neurons project to the Ventral Tegmental Area (VTA) where the release of dynorphin alters the excitability of dopamine neurons to enhance drug reward learning [72], and DR *Pdyn*-expressing neurons promote social deficits during drug withdrawal via their Nucleus Accumbens projections [73]. Our findings reveal biphasic regulation of *Pdyn*, with increased expression following acute SSRI treatment and subsequent decrease after chronic administration. This initial upregulation may contribute to the documented adverse effects during early SSRI treatment, including increased anxiety and suicide risk [74, 75].

The neuropeptide-coding gene *Trh* showed opposing temporal regulation compared to *Pdyn*, with a trend for downregulation after acute treatment and robust upregulation following chronic SSRI administration. While thyrotropin-releasing hormone (TRH) is primarily known for its role in the hypothalamic–pituitary–thyroid axis, it is also expressed in limbic regions, including the hippocampus, amygdala, and lateral DR. TRH signals through two receptors (TRHR1 and TRHR2) expressed throughout these regions, suggesting broader functions beyond endocrine regulation. Evidence supports TRH involvement in cognitive and emotional processing [76], with both TRHR1 and TRHR2 knockout mice exhibiting depression- and anxiety-like behaviors [77, 78]. Clinically, depression is associated with blunted TRH response and hypothyroidism [76], though the distinct roles of region-specific TRH systems remain unclear. Whole-brain projection tracing of *Trh*-expressing DR neurons revealed subcortical targeting to the thalamus and hypothalamus, as well as the bed nucleus of the stria terminalis (BNST) and posterior amygdala [15]. Our observation that chronic SSRI treatment upregulates *Trh* expression in the lateral DR suggests future studies to investigate functional antidepressant effects of this molecular subpopulation.

Our findings contribute to previous studies showing 5-HT1A receptor adaptation over the course of SSRI treatment, with *Htr1a* showing upregulation following acute treatment and downregulation after chronic administration in the transcriptomic data. Spatial transcriptomics measurements of gene expression reflect aggregate transcript abundance across multiple cells per spot, representing the combined effect of both the number of expressing cells and expression levels within those cells. Thus, our data cannot definitively distinguish between reduced expression per cell versus fewer expressing cells. Notably, our activity analysis revealed spatial heterogeneity, with chronic treatment reducing activity specifically in the midline DR subcluster (Figure S4D), suggesting that autoreceptor-mediated regulation may vary across serotonergic subpopulations. These transcriptional changes present an interesting contrast with previous electrophysiological and immunohistochemical studies, which showed acute SSRI treatment initially decreases serotonergic neuron activity before returning to baseline during chronic treatment, with variable region-dependent effects [22, 79–81]. This apparent discrepancy might be explained in several ways. First, our spatial transcriptomics data represents mini-bulk measurements, potentially capturing SSRI-induced changes in non-serotonergic neurons within the analyzed regions. Alternatively, distinct serotonergic subtypes may respond differently to SSRI treatment, with some populations showing decreased activity while others maintain or increase their firing rates.

Importantly, *Htr1a* regulation shows regional heterogeneity across the brain, with SSRI treatment producing distinct effects in the DR versus forebrain target regions. While we observed downregulation in the DR consistent with autoreceptor adaptation, studies in hippocampus and prefrontal cortex have reported increased *Htr1a* expression following stress, which is attenuated by chronic fluoxetine treatment [82, 83] and the necessity of hippocampal postsynaptic 5-HT1A expression for antidepressant effects [84]. These regional differences likely reflect the distinct functional roles of 5-HT1A receptors as inhibitory autoreceptors in the DR versus pre- and postsynaptic heteroreceptors in forebrain circuits. The delayed therapeutic effects of SSRIs may arise from coordinated adaptations across multiple brain regions. Our DR-focused analysis captures only one component of this distributed network response.

A limitation of the Visium spatial transcriptomic approach is the lack of single-cell resolution. We estimated that each 55 µm diameter sequenced spot contained 9 cells (see Supplementary Methods, Fig. S3F), resulting in averaged gene expression profiles across potentially heterogeneous cell populations. Furthermore, transcriptional changes occurring in rare cell populations or small neuronal subsets may be diluted below detection thresholds. To address these limitations and validate our findings at single-cell resolution, we employed a complementary two-fold approach. First, we performed multiplexed fluorescent ISH for key differentially expressed genes, enabling precise cellular localization and quantification of transcript abundance in individual neurons. This approach allowed us to determine which specific cell types exhibited treatment-induced changes and to generate a comprehensive molecular map spanning the rostro-caudal axis of the DR (Fig. S2). Our ISH analysis confirmed the opposing SSRI-mediated regulation of *Trh* and *Pdyn* expression. However, several other DEGs identified by spatial transcriptomics (*Htr1a*, *Bdnf* and *Vip*) were not validated by ISH, likely reflecting multiple factors including the higher sensitivity of sequencing-based methods for detecting low-abundance transcripts, and potential contributions from non-neuronal cells captured in spatial transcriptomic spots but not analyzed by ISH. Second, to computationally address the cellular heterogeneity within each spatial spot, we performed cell type deconvolution, integrating our spatial data with a published single-cell RNA-seq reference dataset from the DR [16]. This analysis estimated the relative proportions of different cell types contributing to each spot’s expression profile, providing insight into how observed transcriptional changes were driven by specific cell populations or reflected tissue-wide responses to SSRI treatment.

The discordance between spatial transcriptomics detection and serotonergic co-expression for certain DEGs, exemplified by *Bdnf*, illustrates an important difference where spatial transcriptomics captures the integrated response of the entire tissue microenvironment, while single-cell approaches and ISH validation provide cell-type resolution. Spatial transcriptomics and mapping of DEGs to serotonergic subtypes showed that *Bdnf* upregulation following acute SSRI treatment occurs predominantly in non-serotonergic DR neurons. This finding aligns with the neurotrophic hypothesis of antidepressant action, which emphasizes BDNF-TrkB signaling but does not require that BDNF production be restricted to serotonergic neurons [68, 85]. The identification of Bdnf as a network hub gene despite its primarily non-serotonergic expression suggests that SSRI-induced transcriptional responses involve coordinated communication across multiple cell types within the DR, rather than isolated changes within serotonergic neurons alone.

To investigate the temporal dynamics underlying SSRI therapeutic action, we compared acute versus chronic fluoxetine administration, revealing distinct transcriptional programs that emerge over clinically relevant timescales. We deliberately examined SSRI effects in naive, healthy mice to establish baseline pharmacological responses without confounding pre-existing pathology, in order to identify fundamental transcriptional changes induced by serotonin reuptake inhibition. Future studies using depression or stress models will be essential to capture how SSRIs interact with the molecular alterations present in depression. Furthermore, an important limitation of our study is the use of a single acute control group, which does not account for potential habituation effects, such as the effect of innate coping or homeostatic responses to repeated gavage in the chronic treatment group. Oral gavage induces acute stress responses, and repeated stress can alter DR gene expression, including *Htr1a* upregulation [82]. Thus, we cannot definitively dissociate the transcriptomic effects of chronic stress due to handling and habituation from the purely pharmacological effects of chronic fluoxetine. Future studies with chronic vehicle controls will be valuable to rigorously dissociate these mechanisms.

Overall, our analysis reveals spatio-molecular heterogeneity within the DR and provides new insights into region-specific SSRI actions. While our findings support established theories of SSRI mechanisms, including 5-HT1A adaptation and neurotrophic effects, we also uncovered a potential novel neuropeptide-based mechanism involving dynamic regulation of *Pdyn* and *Trh*. The opposing temporal patterns of these neuropeptides may contribute to both the initial adverse effects and subsequent therapeutic benefits of SSRI treatment. The candidate genes, pathways and cell types uncovered pave the way for deeper mechanistic studies to unravel the underpinnings of depression, and reveal novel targets for more specific pharmacological targeting.

## Supplementary information

Supplementary Information

Supplementary Table 1

Supplementary Table 2

Supplementary Table 3

## Data Availability

Data has been deposited at https://doi.org/10.5281/zenodo.14859692 and is also accessible through an interactive app available at https://st-ssri.serve.scilifelab.se/app/st-ssri. Any additional information and code details are available from the lead contacts upon request.
