# Supplementary material for: Effects of SSRIs on the spatial transcriptome of dorsal raphe serotonin neurons
Source: Mol Psychiatry. 2026 May 15;31(10):5607–18. doi: 10.1038/s41380-026-03644-x (PMC13569412; doi:10.1038/s41380-026-03644-x)
Supplement: Supplementary file 1 [file 41380_2026_3644_MOESM1_ESM.pdf]

Effects of SSRIs on the spatial transcriptome of dorsal raphe serotonin neurons

Henningson Charlotta[1], Mlost, Jakub[1], Pollak Dorocic, Iskra[1]

[1] Science for Life Laboratory, Department of Biochemistry and Biophysics, Stockholm University, Sweden

Corresponding author: Pollak Dorocic, Iskra
iskra.pollak@scilifelab.se

This file includes:

- Supplementary figures 1-8
- Supplementary figure legends 1-8
- Supplementary methods
- Supplementary table legends 1-3

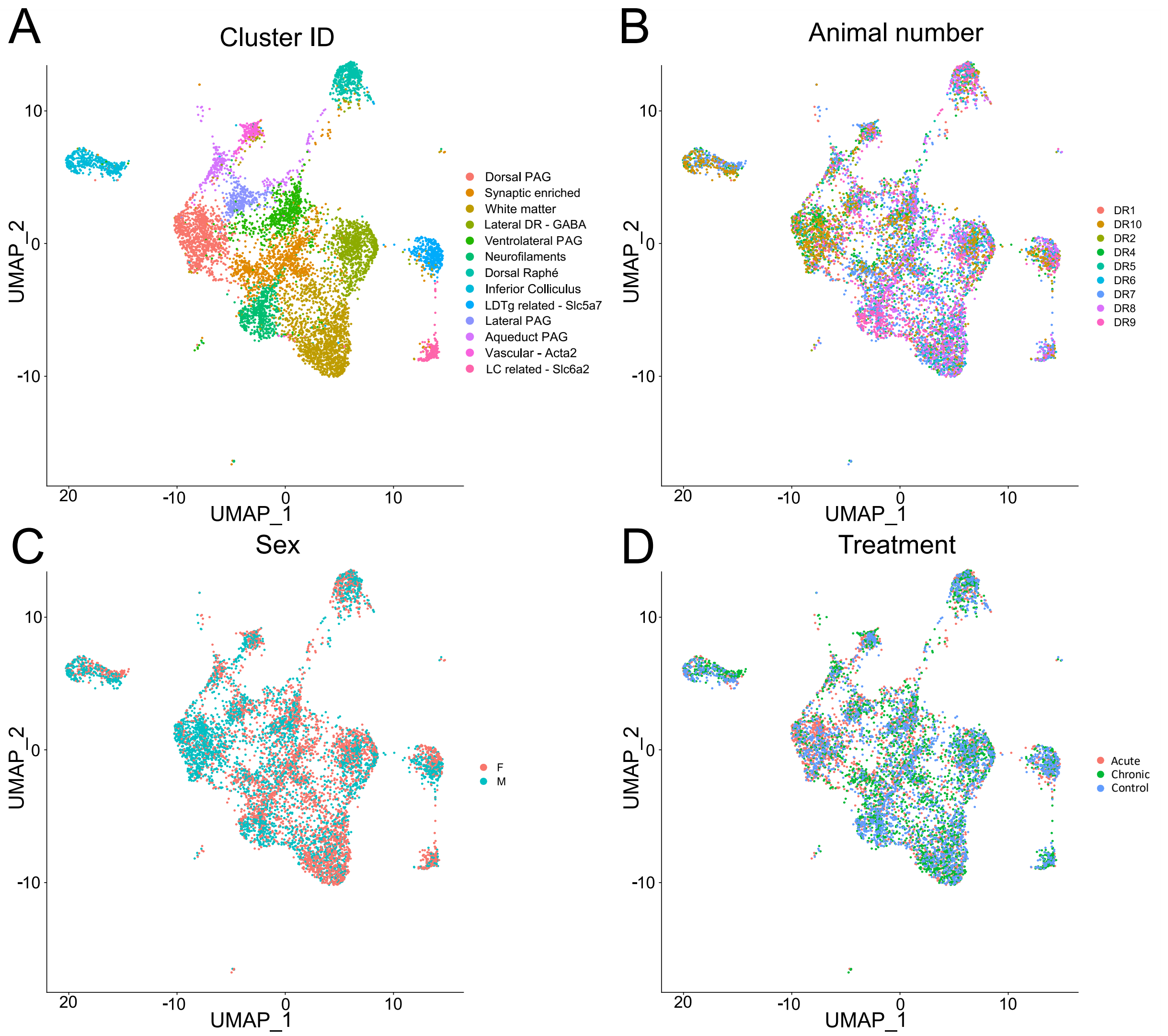

Figure S1. UMAP visualisation of midbrain transcriptomic data show no effect of sample, sex, and treatment in clustering

Lack of the technical- or batch- driven effects on clustering results in UMAP plot (**A**) was visualized by overlaying sample ID from different animals (**B**), their sex (**C**) or treatment group (**D**). Some Sample IDs constitute a larger proportion of clusters located on the edge of the tissue section (eg. Dorsal PAG), which is related to dissection.

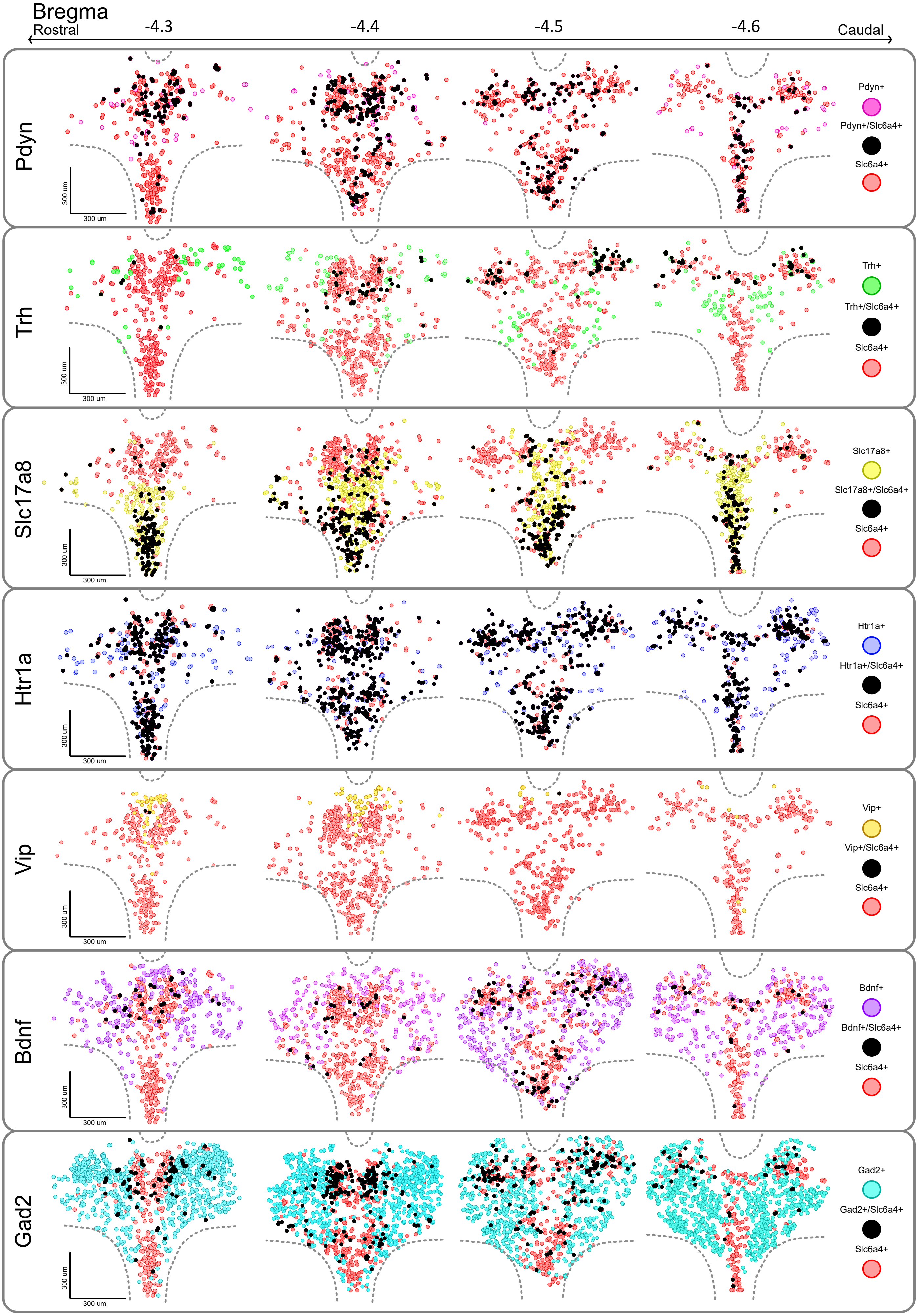

Bregma
Rostral
-4.3
-4.4
-4.5
-4.6
Caudal
Pdyn
Pdyn+
Pdyn+/Slc6a4+
Slc6a4+
Trh
Trh+
Trh+/Slc6a4+
Slc6a4+
Slc17a8
Slc17a8+
Slc17a8+/Slc6a4+
Slc6a4+
Htr1a
Htr1a+
Htr1a+/Slc6a4+
Slc6a4+
Vip
Vip+
Vip+/Slc6a4+
Slc6a4+
Bdnf
Bdnf+
Bdnf+/Slc6a4+
Slc6a4+
Gad2
Gad2+
Gad2+/Slc6a4+
Slc6a4+
300 um
300 um

Figure S2. Spatio-molecular map of serotonin co-expression with other markers in DR

Spatio-molecular maps based on in-situ hybridization imaging (*Pdyn, Trh, Slc17a8, Htr1a, Vip, Bdnf, Gad2*) in the DR, along the rostro-caudal axis (-4.3mm to -4.6mm). Co-expression of each specific marker with *Slc6a4* (SERT) is marked by a black circle (each circle correspond to a single cell), Slc6a4 positive cells lacking marker co-expression are marked in red, and the specific marker cells lacking Slc6a4 expression are shown in their respective color.

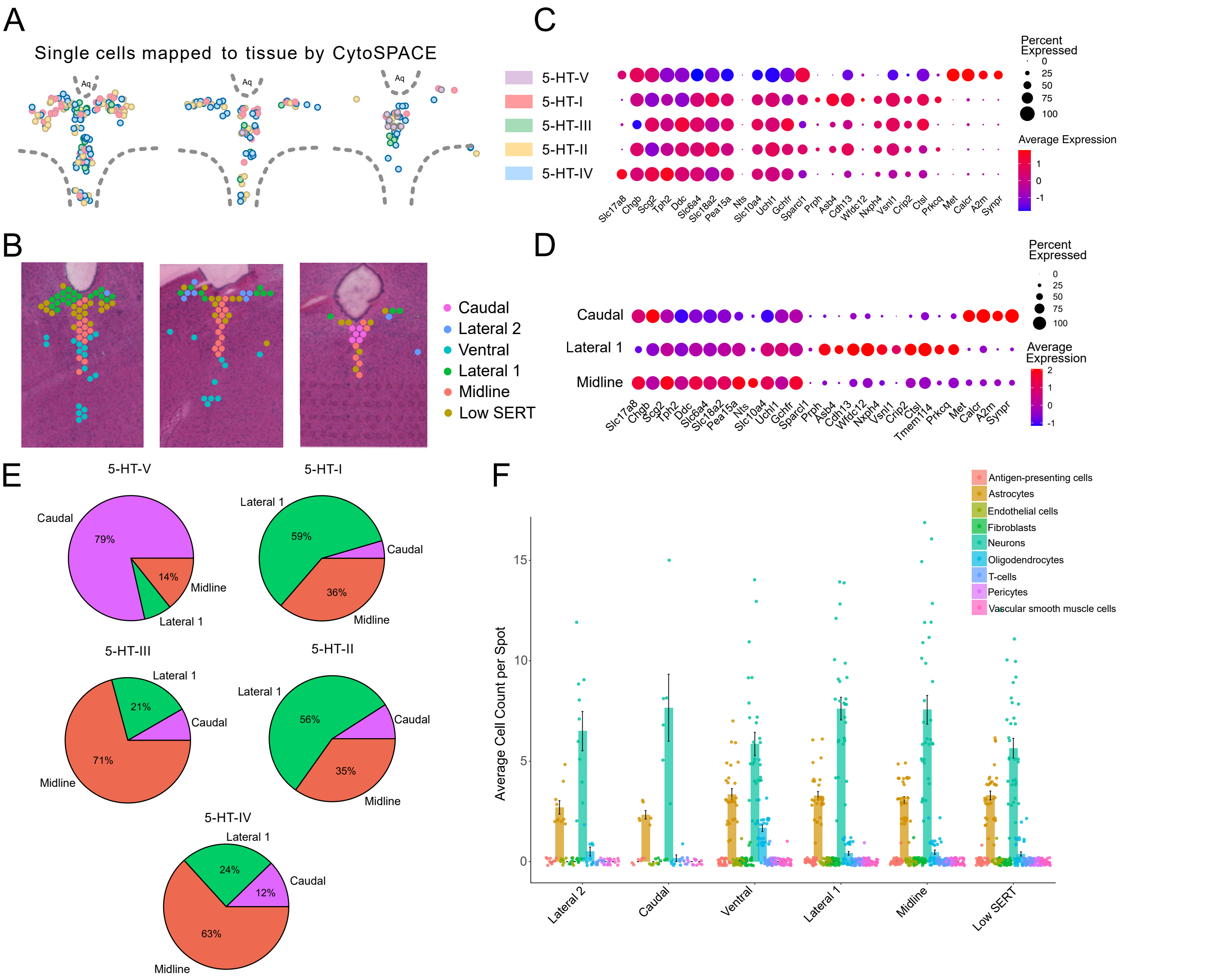

Figure S3. Mapping and deconvolution of serotonin subtypes reveals distinct spatial domains in DR

A. Integration of five molecular subtypes of DR serotonin neurons from published scRNA-seq dataset (5-HT-I through V from Huang, et al. 2019) mapped to spatial location across the rostro-caudal axis, using CytoSPACE computational deconvolution.

B. Spatial transcriptomic clusters overlaid with tissue section, showing spatial localization of each cluster in DR and rostro-caudal expression patterns.

C. Expression of top genes from spatial clusters (Caudal, Lateral 1 and Midline) mapped to five serotonin subtypes (5-HT-I through V). Diameter of the dot corresponds to the percentage of spots in the cluster expressing the gene, colour indicates average expression of the gene.

D. Expression of top genes from spatial clusters (Caudal, Lateral 1 and Midline). Diameter of the dot corresponds to the percentage of spots in the cluster expressing the gene, colour indicates average expression of the gene.

E. The distribution of serotonergic subtypes (5-HT-I through V) quantified across three spatial domains (Caudal, Lateral 1, and Midline), expressed as the percentage of each subtype's total mapped cells within these clusters.

F. Quantification of cell type composition of DR spatial clusters utilizing non-negative regression for inferring single resolution cells to spatial spots using SpaTalk. For each spatial spot in each DR cluster the number of cells belonging to broad cell types (dots) is averaged across the cluster (bar). Error bars represent SEM.

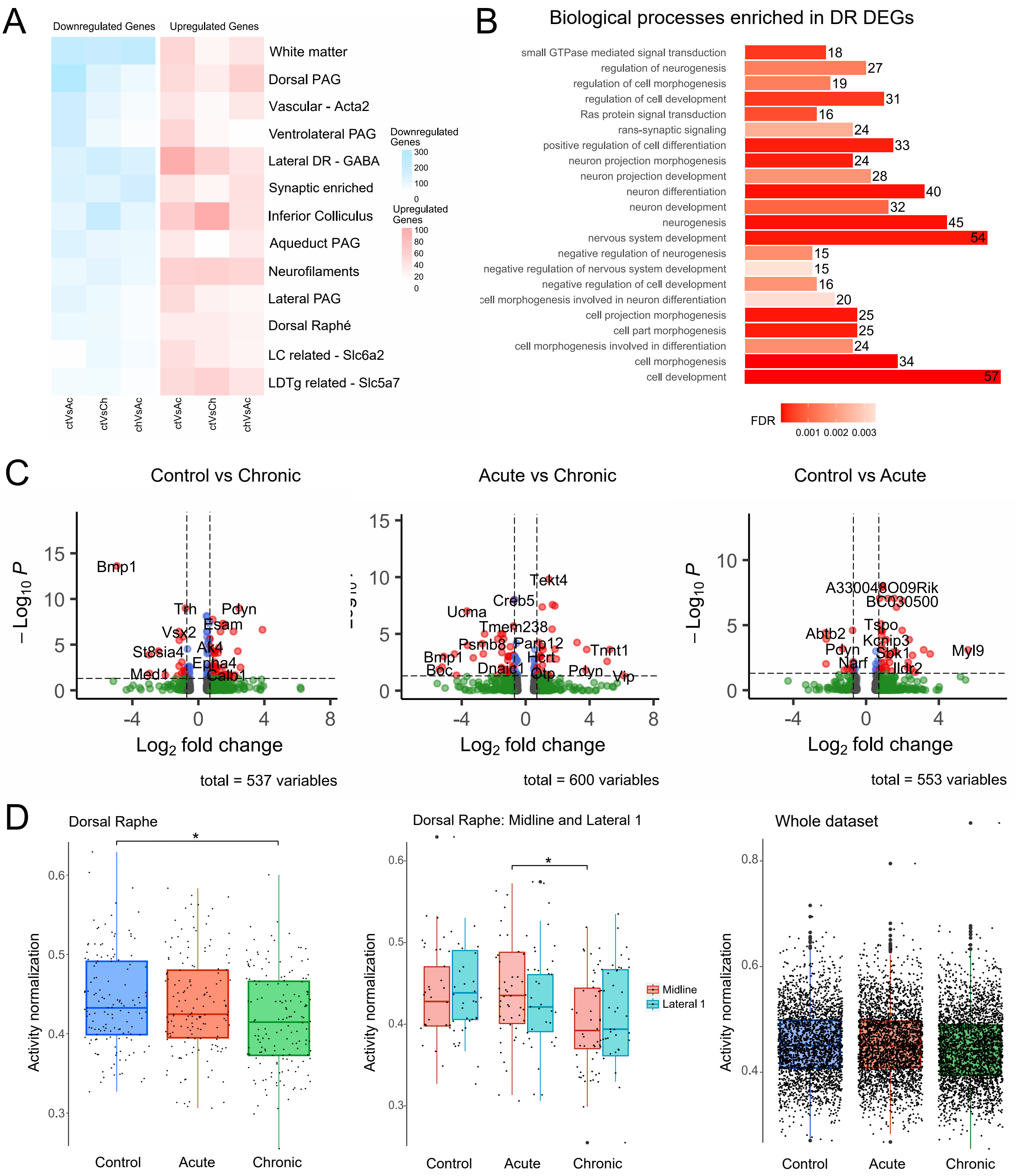

Figure S4. SSRI-induced changes in gene expression and activity in midbrain and DR

A. Heatmap visualization of the number of DEGs in clusters from whole midbrain section, across treatment conditions. The clusters correspond to UMAP in Fig. 1B.

B. Results of gene enrichment analysis in Gene Ontology, Biological Processes of DEGs in the DR across all treatment conditions.

C. Volcano plots representing top up- and down-regulated genes in the DR across treatment conditions. Positive log2 fold change represents lower expression in the first treatment group relative to the second treatment group.

D. Box plot comparing DR cluster neural activity between treatment groups, activity per transcriptomic spot generated by NEUROeSTIMator (one-way ANOVA followed by Tukey multiple comparisons of means, $F(2,443) = 4.101$, $p = 0.0172$*; Control vs Chronic, $p = 0.0229$*), DR Midline vs Lateral 1 cluster (one-way ANOVA followed by Tukey multiple comparisons of means, $F(2,219) = 6.022$, $p = 0.0028$**; Chronic vs Acute, $p = 0.0466$*), and the whole dataset (no difference). Control n = 3, acute n=3, chronic n=3 mice per group. Dots represent individual spots in the ST dataset, $p < 0.05$ = *. Boxplots show data distribution in each group, horizontal line represents the median.

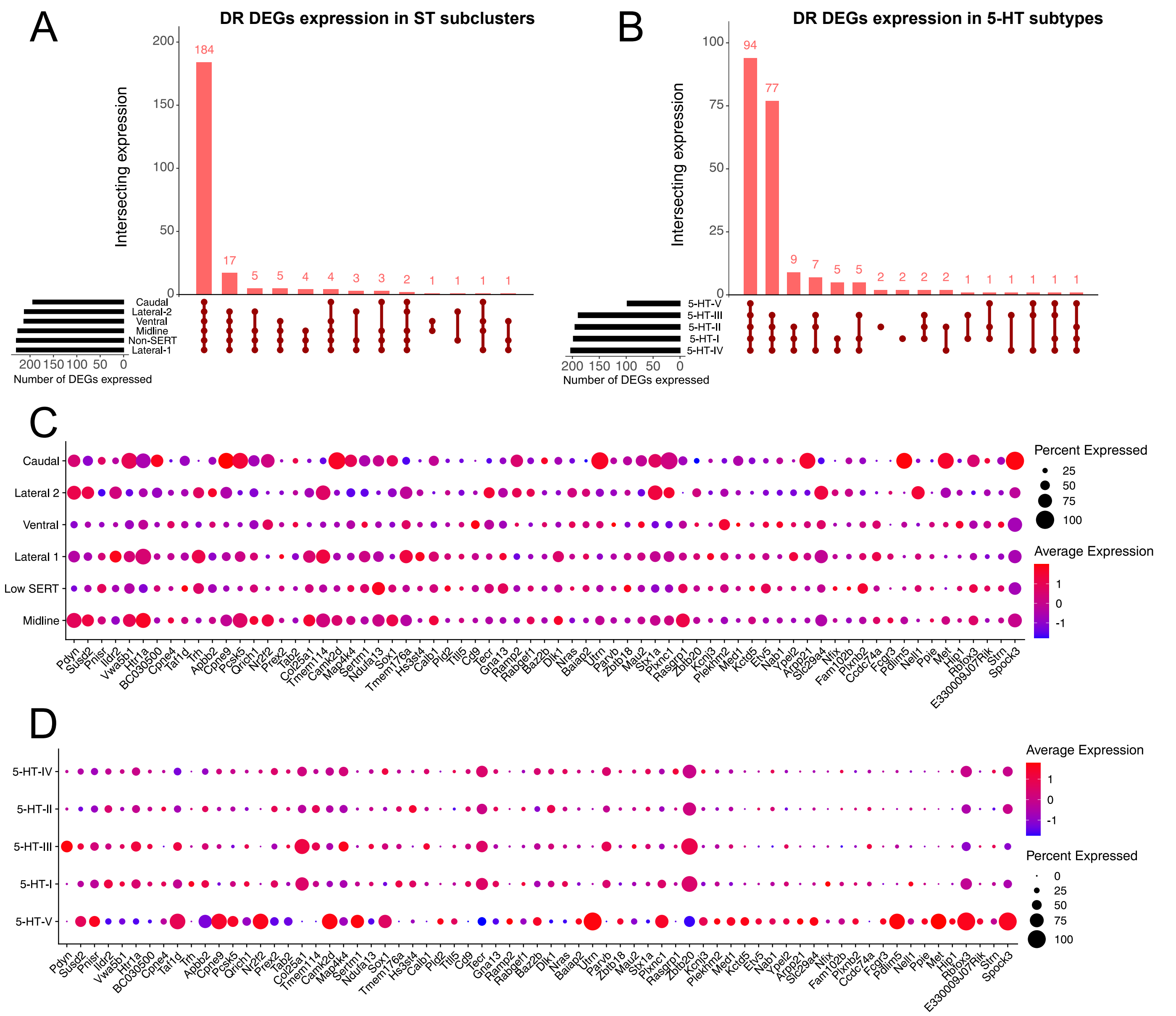

Figure S5. Expression of DR-derived DEGs across spatial subclusters and 5-HT molecular subtypes

A. UpSet plot showing the overlap of DR DEGs expressed across DR spatial subclusters.

B. UpSet plot showing the overlap of DR DEGs expressed across 5-HT neuronal subtypes I-V defined by scRNA-seq dataset (Huang et al., 2019).

C. Dot plot illustrating the expression of DR DEGs in spatial clusters, based on expression in scRNA-seq dataset (Huang et al., 2019), filtered for expression in ≥20% of any 5-HT subtype.

D. Dot plot illustrating the expression of DR DEGs across 5-HT neuronal subtypes, restricted to genes detected in ≥20% of at least one 5-HT subtype.

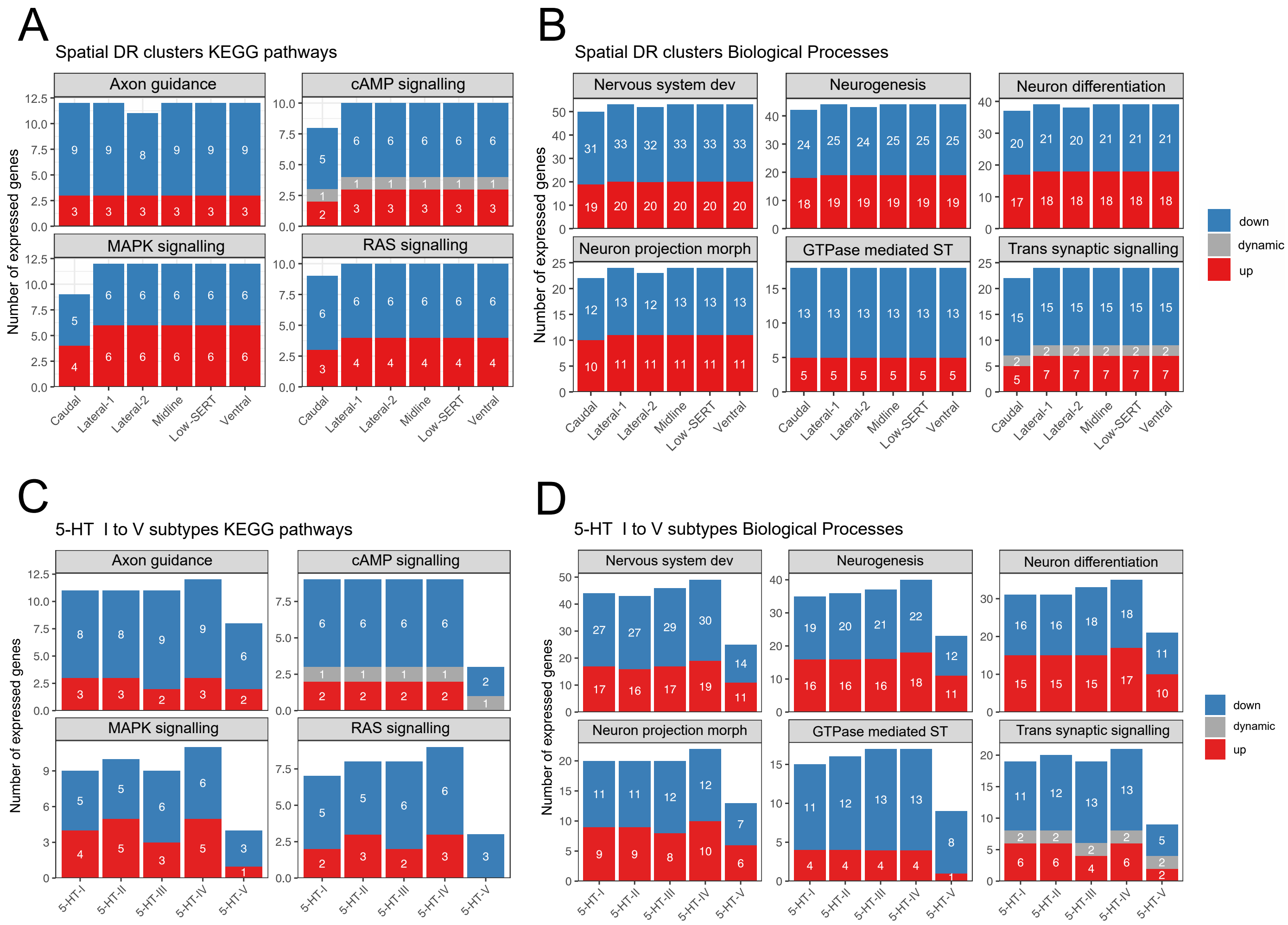

Figure S6. Expression of DR DEGs associated with KEGG Pathways and Biological Processes across DR spatial subclusters and 5-HT molecular subtypes

A. DR spatial subcluster–specific expression of KEGG pathway–associated DEGs.

B. DR spatial subcluster–specific expression of a selection of Biological Process–associated DEGs.

C. 5-HT subtype–specific expression of KEGG pathway–associated DEGs.

D. 5-HT subtype–specific expression of a selection of Biological Process–associated DEGs.

Analysis of 5-HT molecular subtypes I-V as defined by scRNA-seq dataset (Huang et al., 2019). The total number of DEGs associated with KEGG pathways Axonal guidance is 12, RAS signalling is 10, MAPK signalling is 12 and cAMP signalling is 10. The total number of DEGs associated with GO biological processes Nervous system development is 54, Neurogenesis is 45, Neuron differentiation is 40, Neuron projection morphogenesis is 24, Small GTPase mediated signal transduction is 18 and Trans synaptic signalling is 24. Genes are grouped by average up- or down-regulation across treatment progression. Full list of DEGs included in each category can be found in Supplemental Table 3.

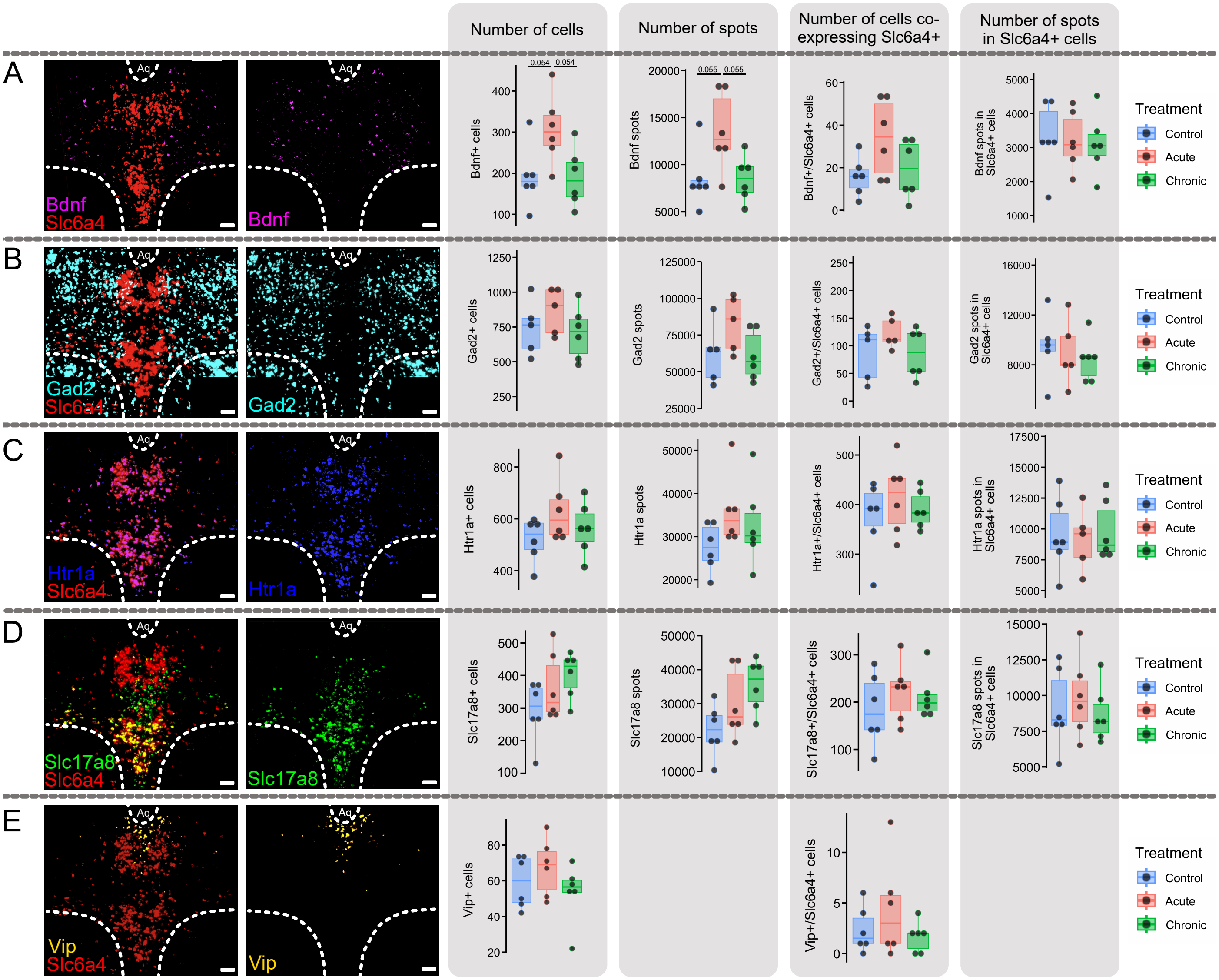

Figure S7. Gene expression of Bdnf, Gad2, Htr1a, Vip, and Slc17a8 in the DR across SSRI treatment

A. Representative images of in-situ hybridization of *Bdnf* and *Slc6a4* co-expression and *Bdnf* in the DR. Box plots comparing treatment groups: sum of *Bdnf*+ cells per section (one-way ANOVA, $F(2,15) = 4.627$, $p = 0.027$*. Pairwise t-test with Holms correction; Acute vs Chronic, $p=0.0539$; Acute vs Control, $p=0.0539$), sum of *Bdnf* spots (transcripts) per section (one-way ANOVA, $F(2,15) = 4.636$, $p = 0.027$*. Pairwise t-test with Holms correction; Acute vs Chronic, $p = 0.0552$; Acute vs Control, $p=0.0552$), sum of cells co-expressing *Bdnf* and *Slc6a4* per section, and sum of *Bdnf* spots in *Slc6a4* cells

B. Representative images of in-situ hybridization of *Gad2* and *Slc6a4* co-expression and *Gad2* in the DR. Box plots comparing treatment groups of sum of *Gad2*+ cells per section, sum of *Gad2* spots (transcripts) per, sum of cells co-expressing *Gad2* & *Slc6a4* per section and sum of *Gad2* spots in *Slc6a4+* cells.

C. Representative images of in-situ hybridization of *Htr1a* and *Slc6a4* co-expression and *Htr1a* in the DR. Box plots comparing treatment groups of sum of *Htr1a*+ cells per section, sum of *Htr1a* spots per slice, sum of cells co-expressing *Htr1a* & *Slc6a4* per and sum of *Htr1a* spots in *Slc6a4* + cells.

D. Representative images of in-situ hybridization of *Slc17a8* and Slc6a4 co-expression and *Slc17a8* in the DR. Box plots comparing treatment groups of sum of *Slc17a8* cells per section, sum of *Slc17a8* spots (transcripts) per slice, sum of cells co-expressing *Slc17a8* and Slc6a4 per and sum of *Slc17a8* spots in Slc6a4 cells.

E. Representative images of in-situ hybridization of *Vip* and *Slc6a4* co-expression and *Vip* in the DR. Box plots comparing treatment groups of sum of *Vip*+ cells per section and sum of cells co-expressing *Vip* & *Slc6a4* per slice. *Vip* spot counts are excluded due to optical crowding of the RNA punctae preventing quantification.

Scale bar = 100μm, control n = 5-6, acute n=6, chronic n=6 mice per group. Boxplots show the data distribution in each group, horizontal line represents the median.

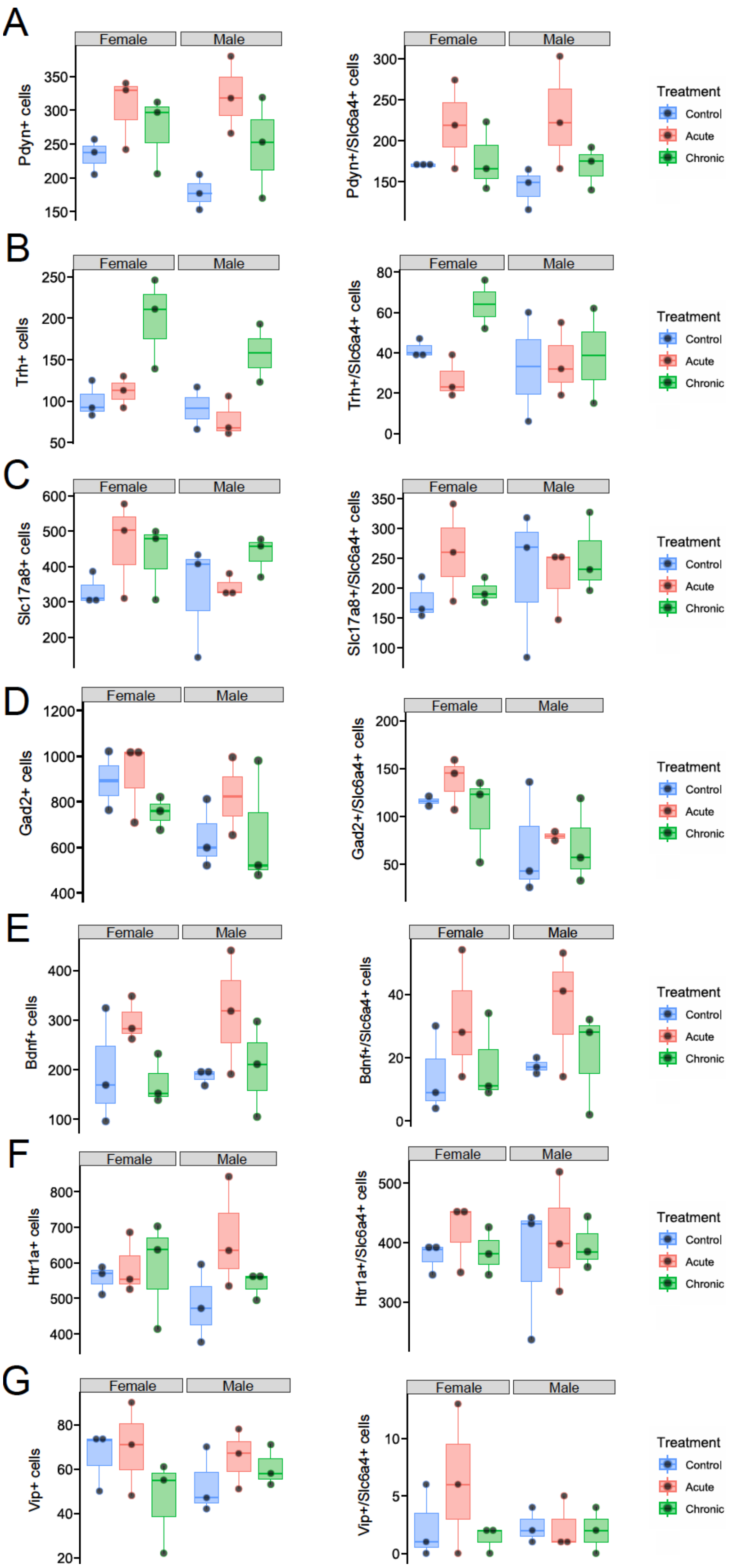

Figure S8. Lack of sex-specific modulation of gene expression in DR by SSRI treatment

In-situ hybridization data show no sex effects are observed due to acute nor chronic SSRI treatment in the expression of *Pdyn*, *Trh*, *Slc17a8*, *Gad2*, *Bdnf*, *Htr1a* and *Vip*.

A. Box plots comparing treatment groups divided by sex: sum of *Pdyn*+ cells per section, sum of cells co-expressing *Pdyn* and *Slc6a4* per section.

B. Box plots comparing treatment groups divided by sex: sum of *Trh*+ cells per section, sum of cells co-expressing *Trh* and *Slc6a4* per section. Control n = 5, acute n=6, chronic n=5/n=4 mice per group.

C. Box plots comparing treatment groups divided by sex: sum of *Slc17a8*+ cells per section, sum of cells co-expressing *Slc17a8* and *Slc6a4* per section.

D. Box plots comparing treatment groups divided by sex: sum of *Gad2*+ cells per section, sum of cells co-expressing *Gad2* and *Slc6a4* per section. Control n = 5, acute n=5, chronic n=6 mice per group.

E. Box plots comparing treatment groups divided by sex: sum of *Bdnf*+ cells per section, sum of cells co-expressing *Bdnf* and *Slc6a4* per section.

F. Box plots comparing treatment groups divided by sex: sum of *Htr1a*+ cells per section, sum of cells co-expressing *Htr1a* and *Slc6a4* per section.

G. Box plots comparing treatment groups divided by sex: sum of *Vip*+ cells per section, sum of cells co-expressing *Vip* and *Slc6a4* per section. Two-way ANOVA using Sidak multiple comparisons. Unless otherwise specified control n = 6, acute n=6, chronic n=6 mice per group.

Boxplots show the data distribution in each group, horizontal line represents the median.

Supplementary Methods

Animal Husbandry

All experiments were conducted using C57BL/6J male and female mice (Charles River Laboratories). Animals were housed under standard laboratory conditions in individually ventilated GM500 cages (Tecniplast), with a constant 12-hour light/dark cycle, temperature maintained at 21 ± 2 °C, and relative humidity between 50–60%. Diet deficient in phytoestrogens (Altromin 1324 variant P, article no 30047) and water ad libitum. Aspen bedding (Tapvei, article no. 2212) was used. The facility operated under Specific Pathogen Free (SPF) conditions in accordance with FELASA guidelines (2014), with weekly cage changes performed in a laminar flow cage-changing station.

Procedures using animals were approved by the Stockholm Ethics committee (Dnr 5966-2021). Animals were divided into three groups no earlier than 56 days of age and were group housed with a maximum of 5 mice per cage. For spatial transcriptomics experiments, 9 mice (5 male, 4 female) were used, for in-situ hybridization 17 mice (9 females, 8 males) were used. Treatment group allocation was determined by cage organization, with all animals housed together in a single cage assigned to the same treatment condition.

Fluoxetine Administration

Fluoxetine hydrochloride (Sigma-Aldrich, F132) was initially dissolved in sterile saline and stored at −20 °C until use. For administration, the solution was diluted to a concentration of 2 mg/mL and delivered via oral gavage at a dose of 10 mg/kg. This dosage was selected to approximate the human equivalent dose range used for the treatment of depression, where typical daily human doses range from 20–80 mg (i.e., 0.33–1.33 mg/kg for a 60 kg adult)[1,2]. Using standard body surface area-based interspecies scaling[2], the human equivalent dose (HED) corresponding to 10 mg/kg in a 20 g mouse was calculated as:

HED (mg/kg) = Animal dose (mg/kg) × (Animal Km / Human Km)

= 10 × ((0.021 / 0.007) / (60 / 1.62)) ≈ 0.81 mg/kg

Importantly, this dose has been validated to achieve therapeutically relevant drug exposure: chronic administration of 10 mg/kg fluoxetine in mice produces steady-state plasma concentrations of 200-300 ng/ml[3], comparable to therapeutic human plasma levels (150-500 ng/ml), and achieves ~80% serotonin transporter occupancy in brain tissue[4,5]. These pharmacokinetic parameters correlate with antidepressant-like behavioral effects emerging after 3-4 weeks of treatment[3,6], mirroring the delayed therapeutic onset observed clinically.

Gavage was performed using soft, flexible feeding tubes to minimize discomfort and avoid tissue injury. Mice in the acute treatment group received a single 10 mg/kg dose, and brain tissue was collected 1.5 hours post-administration, based on prior reports of robust behavioral responses occurring as early as 30 minutes post-dose[7]. Chronic treatment groups received 10 mg/kg fluoxetine daily for 22 consecutive days, with tissue collected 5 hours after the final dose to reduce confounding acute effects. Control animals received saline at equivalent volumes and were sacrificed 1.5 hours post-injection.

Tissue Preparation

Mice were anesthetized with isoflurane and euthanized by cervical dislocation. Brains were rapidly extracted and briefly immersed (<15 s) in ice-cold saline. For spatial transcriptomics, the midbrain was dissected on a pre-cooled metal block (approx. 5 × 5 × 2 mm), while whole brains were used for RNAscope analysis. All tissues were snap frozen in powdered dry ice

and stored at −80 °C until further processing. Instruments and surfaces were cleaned with 70% ethanol and RNase Away (Thermo Scientific, 10666421) before and during dissections. For spatial transcriptomics, midbrains were embedded in O.C.T. compound (VWR, 361603E), sectioned coronally at 10 µm, and placed onto Visium capture areas (3–4 sections per area). For RNAscope, whole brains were sectioned at 20 µm and mounted on SuperFrost+ slides, dried in the cryostat chamber, and stored at −80 °C. The dissected dorsal raphe (DR) regions were localized between bregma −4.3 and −4.7 mm.

Fluorescent In Situ Hybridization

RNAscope HiPlex assays were performed using fresh-frozen 20 µm sections, following the manufacturer's protocol (ACD Bio). In brief, the tissue was fixed in 4% paraformaldehyde (Sigma-Aldrich, 252549) for 60 minutes at room temperature, followed by serial dehydration in 50%, 75%, and two 99% ethanol washes (5 minutes each, Fisher Chemical E/0600DF/F17), then stored in 99% ethanol overnight at −20 °C.

The following day, slides were air-dried and circled with a hydrophobic barrier. Sections were permeabilized with Protease IV (30 min, RT), then incubated at 40 °C with custom probe mixtures (8 target probes, or positive/negative controls) in a humidity-controlled chamber for 2 hours. Three amplification steps (30 min each, 40 °C) followed. To minimize autofluorescence, slices were treated with 3% FFPE reagent mix (30 min, RT), prior to hybridization with HiPlex Fluoro T1–T4 v2 reagents (15 min, 40 °C). After DAPI staining and mounting, the first imaging round was performed.

On the second day, slides were washed, incubated twice with cleaving solution (15 min, RT), and hybridized with HiPlex Fluoro T5–T8 v2 reagents (15 min, 40 °C). The following probes were used: Htr1a-T1, Vip-T2, Bdnf-T3, Pdyn-T4, Trh-T5, Slc6a4-T6, Slc17a8-T7, Gad2-T8.

Microscopy and Image Analysis

Imaging was conducted on a Leica DM6 B upright microscope using a 40× oil immersion objective and LAS X software. Brightfield scans were used to define ROI coordinates. Each ROI was scanned using five fluorescence channels (DAPI, 488, 550, 650, 750 nm), acquiring z-stacks of 8×8 µm per slice over two rounds. Mosaic images were stitched using LAS X's "smooth blend" setting.

Images were processed in Fiji (ImageJ v2.14.0) using Bio-Formats to generate max intensity projections for each round. DAPI-based alignment across rounds was achieved using BigWarp, followed by cell segmentation in QuPath (v0.5.0) with the Stardist model (dsb2018_heavy_augment.pb), applying a 5.0-pixel expansion and 0.5 threshold[8–12].

A region of interest encompassing Slc6a4+ cells in the DR was delineated. Cell identities were assigned via QuPath's object classification, trained per image or dataset. RNA puncta were detected using QuPath's subcellular detection function, with thresholds tailored to background autofluorescence and negative control intensities. Annotations and quantifications were transferred across imaging rounds using the Warpy plugin.

ISH Data Analysis and Statistics

Exported data from both imaging rounds were analyzed in R (v4.2.2). Puncta counts per cell were aggregated and merged using unique cell IDs. Summary tables were generated across animals and slices, reporting total counts per marker and subpopulations. Data normality was assessed; ANOVA was used to test for differences across treatment groups, followed by Holm-adjusted pairwise t-tests if significant ($p < 0.05$). Analysis was performed blind to treatment conditions.

Spatial Transcriptomics

RNA integrity (RIN >8) was confirmed using an Agilent Bioanalyzer. The Visium spatial transcriptomics workflow (10x Genomics) was performed at SciLifeLab, Sweden. Tissue sections were methanol-fixed (−20 °C), stained with hematoxylin and eosin (H&E), and imaged. Enzymatic tissue permeabilization was followed by mRNA capture via poly-A hybridization on spatially barcoded slides. Libraries were constructed per capture area and sequenced.

Manual alignment of histology images to capture areas was done in Loupe Browser (v5.1.0). FASTQ files were processed using Space Ranger (v1.3.0) with the mm10 (GRCh38, 2020-A) reference genome.

Spatial Transcriptomics Data Analysis

Using R (v4.2.2) and Seurat (v4.3.0), spots were filtered to retain those with >1000 genes and <24% mitochondrial content. Mitochondrial, ribosomal, hemoglobin, and sex chromosome genes were excluded. SCTransform normalization (glmGamPoi method, v2 vst flavor) was applied with regression on mitochondrial and ribosomal gene percentages[13–17].

Reciprocal PCA integration (top 3000 features, 30 PCs, k=10) used control samples as anchors. Clustering used the Louvain algorithm (resolution = 0.9), and dimensionality reduction was done via UMAP (20 PCs, 50 neighbors, spread = 2). Clusters not present in ≥2 slices/condition were excluded. A DR cluster (high Tph2 expression) was subclustered (8 PCs, resolution = 0.8).

Differential expression was assessed using FindMarkers() with Wilcoxon Rank Sum test (logfc ≥ 0.5, expression in ≥20% of spots). Only genes passing FDR < 0.05 were considered significant.

Network analysis

To identify key genes in DR affected by SSRI treatment, we created a Protein–Protein Interaction (PPI) Network with the STRING database using the list of all DEGs detected in DR, with significance cutoff value of FDR < 0.05 and minimum expression in at least 20% of the cluster spots. Network was then visualized and analyzed in Cytoscape (3.10.0) [18]. Based on the degree distribution in all three networks, the degree threshold value for hub genes was set to 6. The PPI network incorporates protein interactions known from experiments, text mining, databases, co-expression, co-occurrence, neighbourhood and gene fusion.

Gene enrichment analysis

In order to investigate the potential functional effects of the SSRI treatment in the DR we performed gene enrichment analysis through The Database for Annotation, Visualization and Integrated Discovery (DAVID) bioinformatics [19,20]. We uploaded all DEGs detected in the DR, with significance cutoff value of FDR < 0.05 and minimum expression in at least 20% of the cluster spot, as a list in the DAVID analysis wizard and selected annotation categories KEGG pathways and GO terms Biological processes_5. Expression patterns of DR-derived DEGs, including subsets annotated to KEGG pathways and GO biological processes, were evaluated within Visium DR spatial subclusters and across serotonergic subtypes from the Huang et al., 2019 scRNA-seq dataset[21].

Functional Activity and Cell Type Deconvolution

NEUROeSTIMator v0.1 was used to estimate transcriptional activity from Visium counts. One-way ANOVA followed by Tukey's post hoc tests were performed [22].

Cell-type deconvolution was conducted with SpaTalk (v1.0) CytoSPACE (v1.0.5) using the Huang et al. (2019) single-cell dataset [23–25]. Cell number estimates (n=9) were guided by VistoSeg [26]. All mapped cell types were identified, and serotonin (5-HT) subtypes were isolated for further analyses.

Reproducibility and sample size

Sample sizes were based on previous studies. All experiments included 3 biological replicates per spatial transcriptomics group, and 6 biological replicates per ISH group.

For spatial transcriptomics, exclusion criteria was <1000 genes per spot, > 24% mitochondrial content, or if clusters were present in less than 2 sections per treatment. One section was additionally excluded due to not containing the DR brain region.

For RNAscope, positive-control probes produced robust signal in all included sections, confirming RNA integrity and successful assay performance. Negative-control probes background signal were used collectively to define minimum channel-specific detection thresholds for puncta identification across all images

Exclusion criteria for RNAscope included tissue folding or significant freezing artifacts, uneven hybridization, or regions with markedly reduced or absent signal across large areas of the section, imaging artifacts including high autofluorescence, significant photobleaching, and saturated pixels. Entire sections or individual fluorescence channels were removed when these quality criteria were not met. When possible, additional sections from the same animal were imaged to ensure inclusion of one anatomically matched, high-quality section per animal.

Statistical tests

Spatial transcriptomics data were analyzed using non-parametric tests, with false discovery rate (FDR) correction applied to account for multiple comparisons. Wilcoxon rank-sum tests were used.

NEUROeSTIMator data were analyzed using one-way ANOVA to compare treatment groups. Normality and homogeneity of variance assumptions were verified, and post-hoc comparisons were performed using Tukey's test. All assumptions for parametric testing were satisfied.

For KEGG and GO biological processes Fishers exact test was used with FDR multiple comparison correction.

For RNAscope quantification, differences among treatment groups were evaluated using one-way analysis of variance (ANOVA). Normality and variance homogeneity were confirmed, and post-hoc pairwise comparisons were conducted using t-tests with Holm's correction for multiple testing.

To investigate potential sex-specific effects, two-way ANOVA was performed with treatment and sex as factors. No post-hoc tests were applied, as no significant effects were observed.

References:

1. Altamura, A. C., Moro, A. R. & Percudani, M. Clinical Pharmacokinetics of Fluoxetine. *Clin. Pharmacokinet.* **26**, 201–214 (1994).
2. Nair, A. B. & Jacob, S. A simple practice guide for dose conversion between animals and human. *J Basic Clin Pharm* **7**, 27–31 (2016).
3. Dulawa, S. C., Holick, K. A., Gundersen, B. & Hen, R. Effects of Chronic Fluoxetine in Animal Models of Anxiety and Depression. *Neuropsychopharmacol* **29**, 1321–1330 (2004).
4. Meyer, J. H. *et al.* Serotonin transporter occupancy of five selective serotonin reuptake inhibitors at different doses: an [11C]DASB positron emission tomography study. *Am J Psychiatry* **161**, 826–835 (2004).

5. Hirano, K. *et al.* Relationship between brain serotonin transporter binding, plasma concentration and behavioural effect of selective serotonin reuptake inhibitors. *Br J Pharmacol* **144**, 695–702 (2005).
6. Hodes, G. E., Hill-Smith, T. E. & Lucki, I. Fluoxetine treatment induces dose dependent alterations in depression associated behavior and neural plasticity in female mice. *Neurosci Lett* **484**, 12–16 (2010).
7. Jin, Z. *et al.* Mouse strain differences in SSRI sensitivity correlate with serotonin transporter binding and function. *Sci Rep* **7**, 8631 (2017).
8. Schindelin, J. *et al.* Fiji: an open-source platform for biological-image analysis. *Nat Methods* **9**, 676–682 (2012).
9. Linkert, M. *et al.* Metadata matters: access to image data in the real world. *J Cell Biol* **189**, 777–782 (2010).
10. Bogovic, J. A., Hanslovsky, P., Wong, A. & Saalfeld, S. Robust registration of calcium images by learned contrast synthesis. in *2016 IEEE 13th International Symposium on Biomedical Imaging (ISBI)* 1123–1126 (IEEE, Prague, Czech Republic, 2016). doi:10.1109/ISBI.2016.7493463.
11. Bankhead, P. *et al.* QuPath: Open source software for digital pathology image analysis. *Sci Rep* **7**, 16878 (2017).
12. Stevens, M. *et al.* StarDist Image Segmentation Improves Circulating Tumor Cell Detection. *Cancers (Basel)* **14**, 2916 (2022).
13. Hao, Y. *et al.* Dictionary learning for integrative, multimodal and scalable single-cell analysis. *Nat Biotechnol* https://doi.org/10.1038/s41587-023-01767-y (2023) doi:10.1038/s41587-023-01767-y.
14. Butler, A., Hoffman, P., Smibert, P., Papalexi, E. & Satija, R. Integrating single-cell transcriptomic data across different conditions, technologies, and species. *Nat Biotechnol* **36**, 411–420 (2018).
15. Stuart, T. *et al.* Comprehensive integration of single-cell data. *Cell* **177**, 1888-1902.e21 (2019).
16. Hao, Y. *et al.* Integrated analysis of multimodal single-cell data. *Cell* **184**, 3573-3587.e29 (2021).
17. Satija, R., Farrell, J. A., Gennert, D., Schier, A. F. & Regev, A. Spatial reconstruction of single-cell gene expression data. *Nat Biotechnol* **33**, 495–502 (2015).
18. Shannon, P. *et al.* Cytoscape: A Software Environment for Integrated Models of Biomolecular Interaction Networks. *Genome Res* **13**, 2498–2504 (2003).
19. Bt, S. *et al.* DAVID: a web server for functional enrichment analysis and functional annotation of gene lists (2021 update). *Nucleic acids research* **50**, (2022).
20. Huang, D. W., Sherman, B. T. & Lempicki, R. A. Systematic and integrative analysis of large gene lists using DAVID bioinformatics resources. *Nat Protoc* **4**, 44–57 (2009).
21. Huang, K. W. *et al.* Molecular and anatomical organization of the dorsal raphe nucleus. *eLife* **8**, e46464 (2019).
22. Bahl, E. *et al. NEUROeSTIMator: Using Deep Learning to Quantify Neuronal Activation from Single-Cell and Spatial Transcriptomic Data*. http://biorxiv.org/lookup/doi/10.1101/2022.04.08.487573 (2022) doi:10.1101/2022.04.08.487573.
23. Shao, X. *et al.* Knowledge-graph-based cell-cell communication inference for spatially resolved transcriptomic data with SpaTalk. *Nat Commun* **13**, 4429 (2022).
24. Vahid, M. R. *et al.* High-resolution alignment of single-cell and spatial transcriptomes with CytoSPACE. *Nat Biotechnol* https://doi.org/10.1038/s41587-023-01697-9 (2023) doi:10.1038/s41587-023-01697-9.
25. Huang, K. W. *et al.* Molecular and anatomical organization of the dorsal raphe nucleus. *eLife* **8**, e46464 (2019).
26. Tippani, M. *et al.* VistoSeg: Processing utilities for high-resolution images for spatially resolved transcriptomics data. *Biological Imaging* **3**, e23 (2023).

Supplementary Table Legends

Supplementary Table 1: List of DEGs from Dorsal Raphe cluster, control, acute and chronic fluoxetine administration from Visium spatial transcriptomics.

Supplementary Table 2: Expression of DEGs in Dorsal Raphe spatial subclusters from Visium spatial transcriptomics.

Supplementary Table 3: Expression of DEGs in Dorsal Raphe corresponding to GO biological processes and KEGG pathways enrichment analysis from Visium spatial transcriptomics.
